# Global Transcriptional Response of *Aspergillus niger* to Blocked Active Citrate Export through Deletion of the Exporter Gene

**DOI:** 10.3390/jof7060409

**Published:** 2021-05-23

**Authors:** Thanaporn Laothanachareon, Lyon Bruinsma, Bart Nijsse, Tom Schonewille, Maria Suarez-Diez, Juan Antonio Tamayo-Ramos, Vitor A. P. Martins dos Santos, Peter J. Schaap

**Affiliations:** 1 Laboratory of Systems and Synthetic Biology, Department of Agrotechnology and Food Sciences, Wageningen University & Research, 6708 WE Wageningen, The Netherlands; lyon.bruinsma@wur.nl (L.B.); bart.nijsse@wur.nl (B.N.); tom.schonewille@wur.nl (T.S.); maria.suarezdiez@wur.nl (M.S.-D.); peter.schaap@wur.nl (P.J.S.); 2 Enzyme Technology Laboratory, Biorefinery and Bioproduct Research Group, National Center for Genetic Engineering and Biotechnology, 113 Thailand Science Park, Khlong Luang, Pathumthani 12120, Thailand; 3 International Research Center in Critical Raw Materials-ICCRAM, University of Burgos, 09001 Burgos, Spain; jatramos@ubu.es; 4 LifeGlimmer GmbH, 12163 Berlin, Germany

**Keywords:** *Aspergillus niger*, citrate exporter, *citT* gene, *cexA* gene, transcriptome, citric acid, oxalic acid

## Abstract

*Aspergillus niger* is the major industrial citrate producer worldwide. Export as well as uptake of citric acid are believed to occur by active, proton-dependent, symport systems. Both are major bottlenecks for industrial citrate production. Therefore, we assessed the consequences of deleting the *citT* gene encoding the *A. niger* citrate exporter, effectively blocking active citrate export. We followed the consumption of glucose and citrate as carbon sources, monitored the secretion of organic acids and carried out a thorough transcriptome pathway enrichment analysis. Under controlled cultivation conditions that normally promote citrate secretion, the knock-out strain secreted negligible amounts of citrate. Blocking active citrate export in this way led to a reduced glucose uptake and a reduced expression of high-affinity glucose transporter genes, *mstG* and *mstH*. The glyoxylate shunt was strongly activated and an increased expression of the OAH gene was observed, resulting in a more than two-fold higher concentration of oxalate in the medium. Deletion of *citT* did not affect citrate uptake suggesting that citrate export and citrate uptake are uncoupled from the system.

## 1. Introduction

*Aspergillus niger* is a versatile, industrially competitive fungal chassis due to its excellent performance in the hydrolysis of carbohydrates, and its naturally high secretion capacity of proteins and organic acids. These characteristics make *A. niger* a proficient cell factory for the production of industrial enzymes such as glucoamylases, phytase and lignocellulosic degrading enzymes, and organic acids, especially citric acid [1,2,3]. Currently, up to 99% of the global production of citric acid involves a microbial process, 80% of which use *A. niger* as cell factory [4,5]. In the industrial demands, the high yield and productivity have become a primary requirement. To achieve high yields, media composition and environmental factors such as the available carbon and nitrogen sources, phosphate concentration, pH of the medium, oxygen supply, and the influence of trace elements have been optimized. 

The environmental pH dictates the organic acid profile of wild-type *A. niger*. In synthetic media with glucose as carbon source, substantial amounts of oxalic acid are produced as long as the pH of the culture is 3 or above [6]. At around pH 3, *A. niger* starts producing citric acid while the optimal pH for the accumulation of the tricarboxylic acid is around pH 2 [7,8,9]. The influence of the dissolved oxygen tension (DOT) in the production of citrate has been described too, and is significantly higher than that needed for growth [10]. Moreover, trace elements have a crucial impact on citric acid accumulation [11]. Copper, iron, and/or zinc can enhance the maximum yield of citric acid, whereas manganese strongly represses citric acid formation [12]. 

Owing to the capacity of *A. niger* to produce a variety of valuable organic acids, many attempts have been made towards improving *A. niger* strains to reduce undesired by-products, optimize production yields, and lower manufacturing costs. Nevertheless, the role of membrane transporters, frequently considered to be industrial bottlenecks, are still only partially understood [7,13]. The *A. niger* transportome that governs cellular influx and efflux of nutrients, ions, and metabolites is extensive and complex. In silico analysis of the theoretical *A. niger* proteome with precomputed profile hidden Markov models obtained from the Pfam database [14], showed that the *A. niger* ATCC1015 genome [15] codes for more than 500 transporter proteins, of which some 250 are predicted to be major facilitator super family proteins with a conserved domain architecture related to sugar transport [16]. Very similar results were obtained for the *A. niger* CBS 513.88 genome [17] and the laboratory strain N402 [18] used in this study. 

Recently, there have been reports on the discovery of a citrate transporter gene of *A. niger* (*citT,* alternative name *cexA*), responsible for active export of citric acid over the plasma membrane [19,20,21]. The heterologous expression of the *citT* gene resulted in citric acid secretion in yeast [20,21], whereas its overexpression in *A. niger* itself strongly boosted the secretion of citric acid, yielding for 5-times higher than the parental strain [21]. These results are very promising for the citric acid manufacturing industries. Creating a new strain by overexpressing the *citT* gene becomes an excellent option to powerfully improve productivity. Furthermore, the deletion of this gene opens new avenues for rerouting pathways in *A. niger* to produce other organic acids, since citric acid cannot be secreted outside the fungal cell. Hence, here we further elucidate the role of this transporter under industrially relevant conditions, by setting to assess the consequences of deleting the *citT*. To this end, we followed the consumption of glucose and citrate as carbon sources at low pH and under controlled cultivation conditions. To study uptake, citrate was used as a sole carbon source, while glucose was used to promote citrate secretion. Fermentation profiles were followed by HPLC to monitor consumption of the selected carbon sources and the secretion of organic acids. Transcriptome and pathway enrichment analysis were performed to explore the underlying gene networks by comparing the responses of the wild-type and the knock-out strain under the different conditions tested. We show that the *citT* gene is responsible for active *A. niger* citrate export, but not for citrate uptake. Blocking active citrate export under citrate producing conditions reduces the uptake of glucose, induces the secretion of oxalic acid and activates the glyoxylate shunt. Growth on citrate in addition leads to oxalic acid formation.

## 2. Materials and Methods

### 2.1. Strains Media, Growth, and Fermentation Conditions

Strains: *A. niger* N402 (*cspA1*) [22] and MA169.4 (*cspA1: pyrG378*, *kusA::DR-amdS-DR*) [23] are used as control and recipient strain. *A. niger* strains were maintained on complete medium (CM) at 30 °C. The MA169.4 strain requires 0.82 mM and 10 mM uridine for solid or liquid medium, respectively. 

Standard media: Complete medium (CM): 1 g/L casamino acid, 5 g/L yeast extract, 1% glucose, 20 mL/L ASPA + N (stock solution 50×: NaNO_3_ 297.5 g/L, KCl 26.1 g/L and KH_2_PO_4_ 74.8 g/L at pH 5.5), 1 mL/L Vishniac solution [24], 1 mM MgSO_4_, and adjusted to pH 5.5 [25]. 

Fermentation and screening media: Screening medium (SM): 1.2 g/L NaNO_3_, 0.5 g/L KH_2_PO_4_, 0.2 g/L MgSO_4_·7H_2_O, 40 μL/L Vishniac, adjusted to pH 3.5 and supplemented with carbon source. Mycerium preparation medium (CM-SLZ): 1 g/L casamino acid, 5 g/L yeast extract, 3 g/L (NH_4_)_2_SO_4_, 1 g/L K_2_HPO_4_, 1 g/L KH_2_PO_4_, 0.5 g/L MgSO_4_·7H_2_O, 0.014 mg/L MnSO_4_·5H_2_O, 0.01 mg/L FeCl_2_·6H_2_O, 0.075 µg/L ZnCl_2_, 1 mL/L Vishniac solution, adjusted to pH 5.5, and supplemented with 10 g/L glucose. Citric acid production medium (SLZ): 3 g/L (NH_4_)_2_SO_4_, 1 g/L K_2_HPO_4_, 1 g/L KH_2_PO_4_, 0.5 g/L MgSO_4_·7H_2_O, 0.014 mg/L MnSO_4_·5H_2_O, 0.01 mg/L FeCl_2_·6H_2_O, 0.075 µg/L ZnCl_2_, adjusted to pH 2.5, and supplemented with 120 g/L glucose [26].

Screening of the *ΔcitT* knock-out strains was performed by shake flask experiments. Mycelium was pregrown with 1 × 10^6^ spores/mL in CM at 30 °C, 200 rpm, for 18 h. The mycelium was harvested, washed with sterile water and transferred to SM supplemented with 10 g/L of glucose as carbon sources. The culture was incubated at 30 °C, 200 rpm and the supernatants were taken every 24 h for analysis of the organic acid content by HPLC.

Controlled fermentations were performed in 1-L fermenters with a working volume of 0.75 L. The temperature was fixed at 30 °C. 30% (*v*/*v*) polypropylene glycol (ppg) was added as an anti-foaming agent (500 µL/L at final concentration). The aeration was supplied by sterile compressed air continuously blown through a sparger at a rate of 0.6 slpm and the stirring speed was adjusted at 1000 rpm. 

To study citrate secretion, 1 × 10^6^ spores/mL spores were inoculated in 200 mL CM-SLZ and incubated at 30 °C, 200 rpm, for 18 h. Mycelium was reaped and then rinsed with equal amounts of water and 10 g wet weight (~0.92 g cell dry weight, CDW) of mycelium was transferred to 750 mL of SLZ medium adjusted to pH 2.5 with 120 g/L glucose as carbon source. Methanol (2% (*v*/*v*)) was added as a promotive element for citrate secretion [27]. The pH was controlled and maintained at pH 2.5 by addition of 5 M NaOH. 

To study citrate uptake pre-cultures were prepared in CM and then transferred to 750 mL of SM, supplemented with 10.5 g/L citric acid monohydrate as carbon source. The pH was not controlled. 

Mycelium was collected every 24 h, quickly washed within 30 sec with demineralized water, and then dried with a single-use towel, snap-frozen with liquid nitrogen and stored at −80 °C until further processing. Glucose and citrate consumption, and organic acid production were followed by high-performance liquid chromatography analysis (HPLC) as described previously [28].

### 2.2. Construction and Initial Screening of ΔcitT Knock-Out Strains for Citrate Secretion 

*A. niger* citrate exporter knock-out strains were constructed using the split-marker approach [29]. The *citT* gene was deleted from the MA169.4 genome (isogenic with N402), which is defective in the Non-Homologous End-Joining (NHEJ) pathway through a transiently silenced *kusA* gene [30]. A protoplast-mediated transformation of *A. niger* MA169.4 was performed by adapting the protocol of Arentshorst et al. [25] as described in Laothanachareon et al. [18]. Lysing enzymes from *Trichoderma harzianum* (Sigma) were used for protoplast formation (400 mg enzymes per g mycelium). The experimental steps representing the construction of the knock-out strains can be found in Supplementary Figure S1 online. Correct deletion of the gene was confirmed with PCR and complete genome sequencing of a selected knock-out strain. PCR primers used are in Supplementary Table S1 online.

For initial screening of knock-out transformant strains, shake flasks were used. The control strain N402 and the three *ΔcitT* candidates were pre-cultured in CM containing 1 × 10^6^ spores/mL at 30 °C and 200 rpm. After 18 h, mycelium was harvested and then rinsed with equal amounts of sterile water (approx. 200 mL). Mycelium (5 g wet weight) was transferred to 200 mL SM medium supplemented with 18 g/L glucose or 10.5 g/L citrate as carbon sources, and then incubated at 30 °C, 200 rpm. Supernatant was collected every 24 h to determine the amount of carbon source consumption and organic acid production by HPLC. After screening, one *ΔcitT* strain was chosen, completely sequenced, and used in fermentation for determining *ΔcitT* acid profiles and preparing samples for RNA sequencing.

### 2.3. Genome Sequencing of the citT Mutant, Comparative Genomics and Bioinformatics 

Whole genome sequencing of the selected strain *ΔcitT* strain was performed by Novogene using Illumina HiSeq (300 bp inserts library with 150 bp paired-end sequencing). Two strategies were used to confirm the integrity of the selected strain. Read mapping against *A. niger* N402 genome using STAR v2.5.0c [31] to establish that there was a complete and clean deletion of the *citT* gene and assembly the HiSeq reads into contigs using a de novo IDBA_UD [32]-based assembly pipeline showing that the *citT* gene at the genomic position was replaced by a single copy of the *A. oryzae pyrG* gene. 

Comparative genomics of strain N402 and citrate hyper-producing strain H915-1 was done with SibeSibelia [33] and a direct read mapping of N402 Illumina HiSeq reads [18] against the H915-1 genome. 

The raw sequencing data (DNA and RNA) have been submitted to the European Nucleotide Archive (ENA) and can be found under the accession number ERS3465414.

### 2.4. Transcriptome and Pathway Enrichment Analysis

Total RNA of *A. niger* was isolated as described in Sloothaak et al. [34]. The quality and quantity of the RNA were assessed by Qsep100^TM^ analyzer (BiOptic) and samples with a quality of 6 or more were selected for RNA sequencing analysis. Total RNA was sent to Novogene Bioinformatics Technology Co. Ltd., (Beijing, China) for whole transcriptome shotgun sequencing. 

RNA seq data processing: Read mapping against *A. niger* N402 genome was performed using STAR v2.5.0c [31]. Gene coverage calculations were performed using BEDTools v2.17.0 [35], and subsequently normalized for the respective library sizes. Differential expression analysis was performed using the R package edgeR [36]. RNAseq normalization and differential expression was performed simultaneously for each comparison. Genes with a count per million (CPM) ≥ 1 in at least two samples were considered to be expressed and kept for further analysis. Trimmed mean of M-values normalization was performed as implemented in the R package edgeR. *p*-values were corrected for multiple testing using the Benjamini–Hochberg procedure. The terms “differentially expressed” and “overexpressed” refer to differences in read counts per CDS, and denote a fold change ≥ 1.5 (FDR ≤ 0.05). 

Enrichment analyses were performed using the hypergeometric function to model the probability density using the “phyper” function from the R package stats [37]. Two types of analysis were performed: KEGG pathway and GO enrichment [38,39]. For the hypergeometric test we considered the universe size, N, to be the total number of EC numbers in all pathways in the genome, m is the number of successes in this universe and is defined as the number of EC numbers in the corresponding pathway in the genome, k and x are the sample size and the number of successes in the sample (or considered gene subset) respectively. Enrichments with a *p*-value lower than 0.05 were considered significant. GO enrichment analyses were performed with Bingo [40].

## 3. Results

### 3.1. Genome Comparison between Strain N402 and the Citrate-Hyperproducing Strain H915-1

Historically, *A. niger* strains have been characterized and individually labeled as efficient producers of a specific organic acid. For instance, strain ATCC 1015 is considered to be a citric-acid-producing strain [15], whereas strain N400, the parent strain of laboratory strain N402 used in this study, was originally characterized as an efficient gluconic acid producer [41]. Nevertheless, an oxaloacetate acetylhydrolase mutant of the N402 strain can produce up to 90 g citric acid from 140 g sucrose in 10 days [6], and a previously reported genome comparison revealed that its genome and that of strain ATCC 1015 are practically identical [18]. 

Recently, the genome of the extensively mutagenized industrial citric acid hyperproducer H915-1 became available [42]. This strain, which can achieve a citrate titer of 157 g/L and a yield of 0.98 g/g total sugar, contains several missense mutations in different proteins involved in citrate synthesis [42] that make it a hyperproducer. Nonetheless, apart from the reported missense mutations, and several genome rearrangements and deletions in H915-1, the genomes of *A. niger* N402 and H915-1 show an overall identity at nucleotide level of more than 98% (see Supplementary Figure S2 online). Based on RNAseq data, comparing the growth and the citrate accumulation phase of the fermentation, Yin et al. [42], reported a list of transporter genes up-regulated during the citrate production phase. This shortlist was proposed to contain candidate citrate transporters, but did not include the *citT* gene first discovered by Odoni et al. [19,20]. 

A single copy of the *citT* gene is present in both strains. For the *citT* gene we did not observe any nucleotide changes between strains H915-1 and N402, and when the 1000 bp upstream and downstream regions were included in the analysis only a single nucleotide change was observed located downstream of the gene. Since there was no notable change in transcriptional activity of the *citT* gene between the growth and production phase in strain H915-1, to study the role of the transporter in the present study three contrasting conditions were compared: (i) citrate producing conditions, (ii) the effect of blocking active export under citrate producing conditions through deletion of the *citT* gene, (iii) and growth on citrate as carbon source. 

### 3.2. Transcriptional Regulation of the Citrate Exporter Gene 

**Figure 1:**
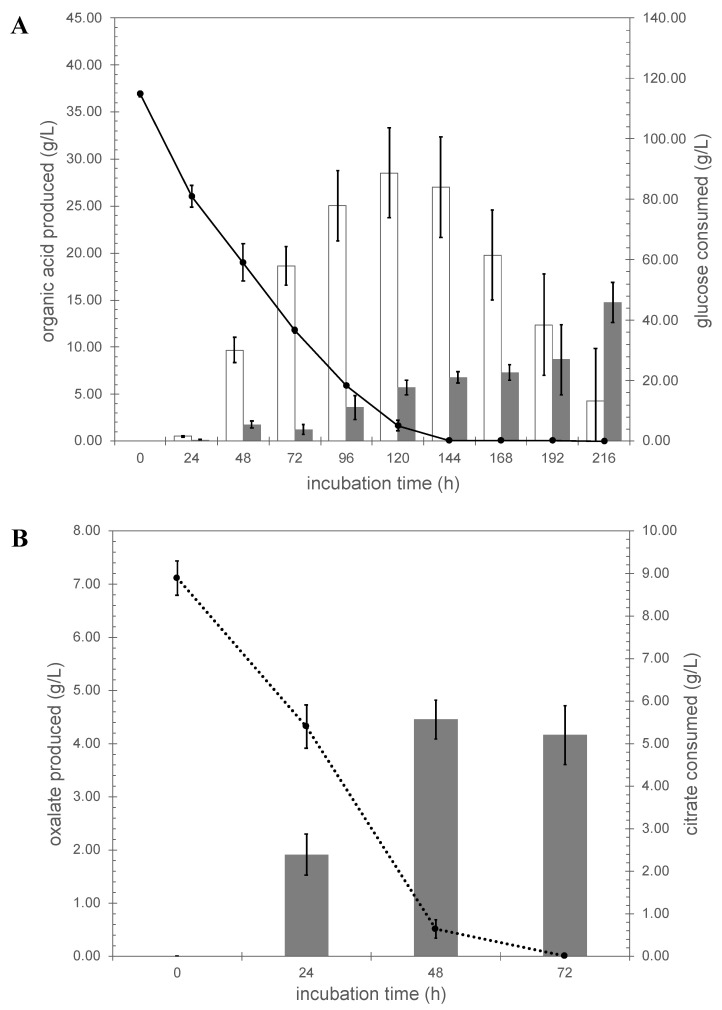
Organic acid profiles of *A. niger* N402 grown in glucose and citrate Panel (**A**), glucose; white bar, amount of citrate produced, gray bar, amount of oxalate produced, dotted line glucose consumed. Panel (**B**), citrate; gray bar, amount of oxalate produced, dotted line is citrate consumed.

*A. niger* N402 was grown in triplicate using controlled fermentation conditions, maintaining the pH at 2.5. This pH was chosen because it is low enough to enable monitoring the secretion of both citric and oxalic acid and to suppress extracellular gluconic acid formation, and high enough to suppress oxalate decarboxylase activity [43]. To observe a significant production of citric acid, fresh mycelium pregrown in CM-SLZ was transferred to SLZ medium with an initial glucose concentration of 120 g/L. Using these fermentation conditions, biomass increased from 1 g/L cell dry weight (CDW) to approximately 15 g/L CDW (see Supplementary Table S2 online). Citric acid was secreted and, under the specific conditions applied, a maximal citrate concentration of 28.52 ± 4.78 g/L was obtained after 120 h of fermentation (Figure 1A). After this timepoint, glucose was depleted from the medium and the cultures started to consume the previously produced citrate. Besides citric acid, oxalic acid was detected in the culture medium, and a final concentration of 15 g/L was obtained after 216 h of fermentation, indicating that oxalic acid production continued after depletion of glucose.

When fresh mycelium pregrown in CM-SLZ was transferred to SLZ medium with citrate as sole carbon source, strain N402 was able to assimilate the citrate, albeit with a much lower efficiency and the initial 9 g/L of citrate in the medium was depleted within 72 h (Figure 1B) while the biomass increased from 1.0 g/L to 1.4 g/L in 72 h (see Supplementary Table S1 online). Upon growth on citrate as a sole carbon source a significant amount of oxalic acid was produced, amounting up to 5 g/L in 48 h (Figure 1B).

**Figure 2:**
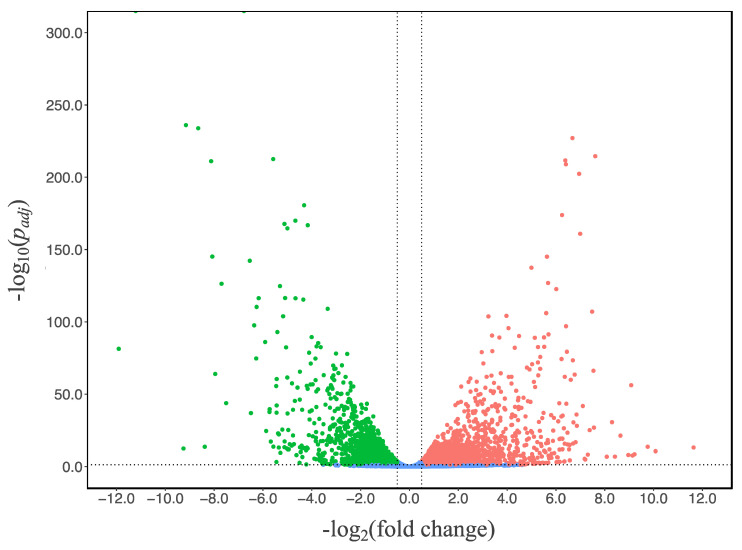
Volcano plot of gene transcript levels of *A. niger* N402 grown in citrate medium using the data of glucose medium as reference. The regions between the dotted lines indicate the non-significant data.

To study the transcriptional response of *A. niger*, we compared the transcriptional landscapes (RNAseq) of the N402 strain grown in glucose and citrate at the 48-h timepoint, which was the early phase of citric acid secretion without consumption. RNA expression data were obtained in triplicate and statistically analyzed using the citric-acid-producing condition as reference. Using a threshold log_2_ (fold change) (log_2_(FC)) value of ≥0.50 or ≤−0.50 and *p_adj_* value below 0.05, 2257 of the 11,187 genes were significantly up-regulated while 2217 genes were down-regulated (Figure 2 and Supplementary Data S1 in sheet N402_citrate_vs_glucose online). 

The CitT exporter protein belongs to Major Facilitator Superfamily (MFS) [19,20]. From the 336 putative MFS encoding genes contained in the genome of *A. niger* N402, 78 and 87 of them were significantly up- and down-regulated in citrate condition compared to glucose (as reference), respectively (Supplementary Data S1 in sheet N402_MFS online). By investigating the list of MFS genes down-regulated upon growth on citrate, a strong down-regulation of the *citT* gene (ATCC64974_65440, corresponding protein id SPB48923.1) was observed with log_2_(FC) at −6.777 (Table 1). The hundred-fold down-regulation suggested that the citrate exporter protein played no role in citrate uptake and prompted us to further study the consequences of blocking active export under otherwise citric-acid-producing conditions.

### 3.3. In Vivo Validation of the A. niger Gene Encoding the Citrate Exporter

To ascertain the functional role of the *citT* gene as the powerfully active export of citrate in its native host, the knock-out of the endogenous *A. niger citT* gene was performed by replacing the gene with the *A. oryzae pyrG* gene as a selection marker. Three independent knock-out strains were selected for further study.

First, growth of the *ΔcitT* strains were monitored on minimal medium (MM) plates supplemented with glucose as carbon source (see Supplementary Figure S3 online). In comparison to the control strain N402, there was no obvious phenotype change. Moreover, the *ΔcitT* strains were able to grow normally on plates containing citrate as sole carbon source, indicating that the deletion of *citT* does not have an impact on citrate import. 

**Figure 3:**
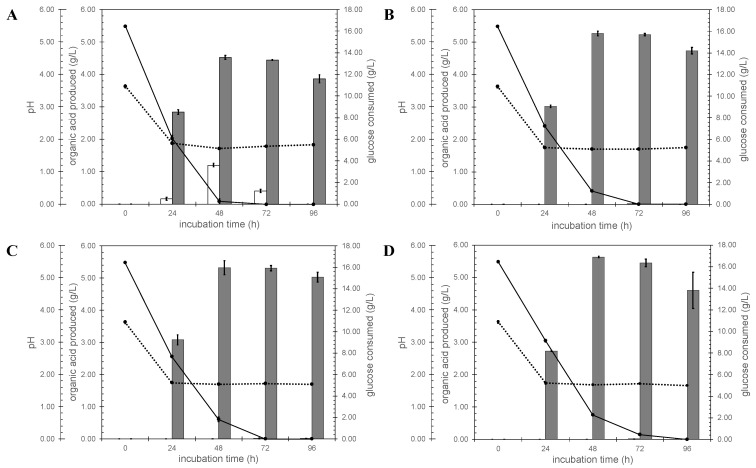
Validation of *A. niger citT* knock-out strains. White bar, the amount of citrate produced; gray bar represents the amount of oxalate produced. Solid line shows glucose concentration and the dotted line corresponds to the culture pH. (**A**) *A. niger* N402 control strain, (**B**) *ΔcitT1.1* (**C**) *ΔcitT2.1* and (**D**) *ΔcitT4.1*.

The *A. niger ΔcitT* strains were then tested for their ability to secrete citrate in shake flask cultures. Glucose consumption, organic acid concentrations and pH were followed in time (Figure 3). All strains acidified the medium. When the N402 control strain was used, the pH dropped from 3.63 to 1.72 in 48 h, and then increased slightly to 1.83 after 96 h of growth (Figure 3A). When the *ΔcitT* strains were used instead, the pH dropped to 1.70 after 48 h of growth and then the pH remained constant for the next 48 h (Figure 3B–D). Besides the formation of biomass, the *ΔcitT* strains secreted only oxalic acid, while the control strain (N402) also secreted citrate. Under these conditions, the highest amount of citrate detected was 1.204 ± 0.053 g/L, at the 48 h timepoint in the N402 strain. After this time point, the glucose was depleted, and the secreted citrate was consumed, which may also explain the slight increase in pH of the N402 strain cultures at the later time points.

These results reconfirmed that the CitT protein can facilitate active *A. niger* citrate export, and that knocking out the *citT* gene significantly impairs the ability of this fungus to secrete citric acid. To further understand the physiological and molecular consequences of a *citT* deletion on active citrate transport, we sequenced the genome of one of the knock-out strains confirming the integrity of this strain and a correct *citT* deletion and used the controlled fermentation conditions previously employed to study the consequences of blocking active citrate transport under citrate production promoting conditions by HPLC and RNAseq.

### 3.4. Organic Acids Production by A. niger Strains N402 and ΔcitT

**Figure 4:**
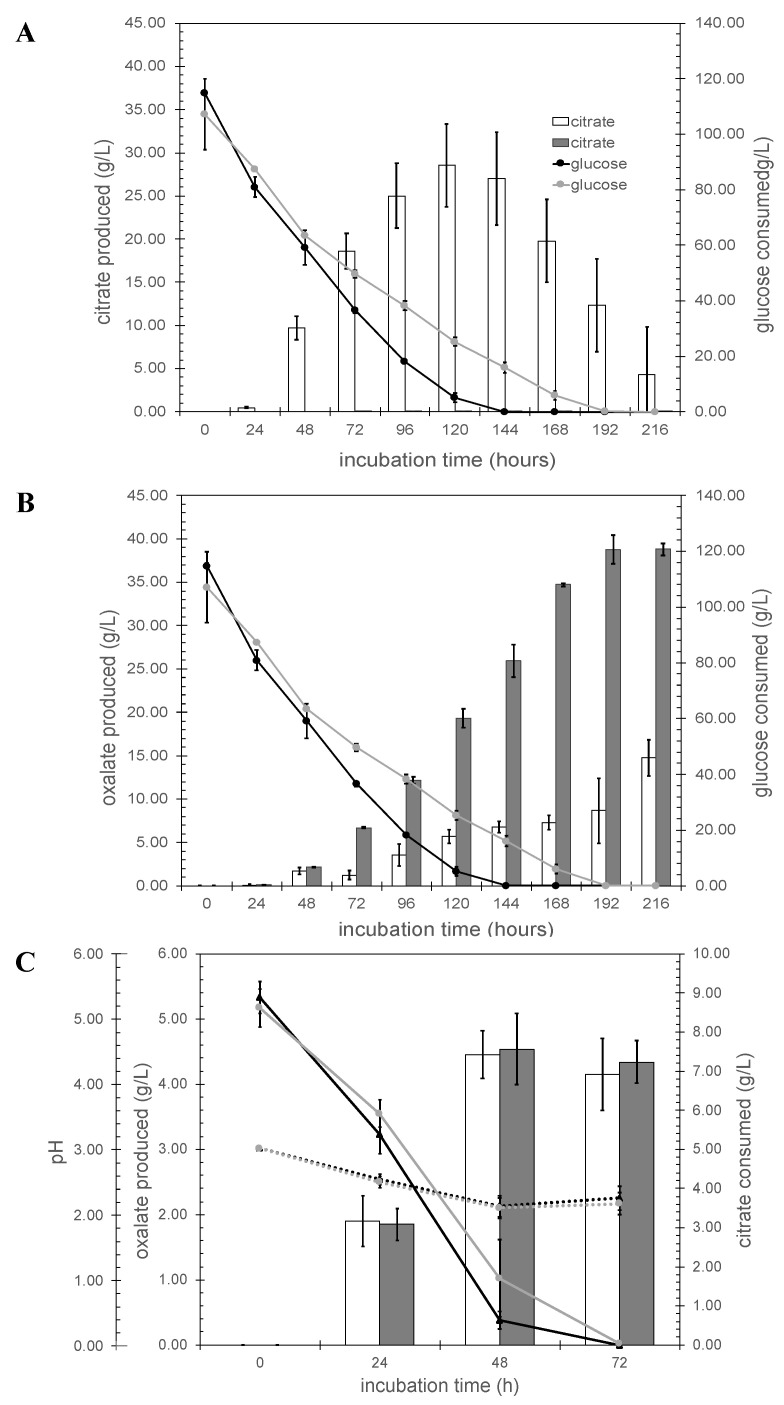
Organic acid production profiles under controlled fermentation conditions. Panel A and B in glucose at pH 2.5. Bar graphs represent the concentration of the citrate (**A**), and oxalate (**B**) formed. Open bars; N402, gray bars; *ΔcitT*. Lines indicate glucose concentration. (**C**) Growth in citrate medium: bar graph represents oxalate concentrations in citrate medium, open bars; N402, gray bars; *ΔcitT.* Solid lines indicate citrate consumed, dotted lines indicate the pH.

Although the specific function of the *citT* gene had been described, the gene networks related to this *citT* gene (and hence, their interplay and role under industrially relevant conditions) are not well-known. Thus, the results herein obtained provide insight into the regulating networks of the *citT* gene. Using controlled fermentation conditions and glucose as carbon source with the control strain, three major organic acids, citrate, oxalate, and fumarate could be detected as extracellular metabolites while only two, oxalate and fumarate, were detected with the *ΔcitT* strain (Figure 4). The maximal citrate titer produced with the *ΔcitT* strain under the conditions applied was 0.10 ± 0.04 g/L compared to a maximal titer of 28.52 ± 4.78 g/L at 120 h with the wild-type strain (Figure 4A). Blocking active citrate transport in this way caused two main side effects; (i) the glucose consumption rate of the *ΔcitT* strain was lower than the control strain, and (ii) oxalic acid was the main product of the *ΔcitT* strain (Figure 4B). The *ΔcitT* strain started secreting oxalic acid already within the first 24 h reaching a titer of 0.132 ± 0.002 g/L. At the 72 h timepoint the reduced glucose uptake of the *ΔcitT* became apparent and the strain also accumulated 5 times more oxalic acid than the control strain (6.69 ± 0.08 g/L vs. 1.24 ± 0.54 g/L). After 216 h, a maximum titer for oxalate of 38.83 ± 0.69 g/L was obtained, matching the solubility of sodium oxalate in water at 30 °C, while the control strain reached a maximum titer of 14.75 ± 2.11 g/L. In addition to citrate and oxalate, fumaric acid was detected during the first 24 h of fermentation, with production levels of 0.72 ± 0.09 g/L for the *ΔcitT* strain and 0.58 ± 0.09 g/L for the control strain. Afterwards, the concentration of fumarate gradually decreased in both cases, not being detected after 144 h in the culture supernatant of the control strain, and after 216 h in the case of the *ΔcitT* strain culture supernatant (see Supplementary Figure S4 online).

When the carbon source was changed to citrate, both strains secreted oxalic acid in comparable amounts (*ΔcitT*: 4.54 ± 0.55 g/L, control strain: 4.45 ± 0.37 g/ at 48 h), although we noted that the citrate consumption rate of the *ΔcitT* strain appeared to be somewhat lower than that of the wild-type strain (Figure 4C).

### 3.5. Transcriptome and Pathway Enrichment Analysis

To gain further insight in the molecular consequences of blocking active citrate export, the 48-h timepoint was used to explore the transcriptional landscape of the *citT* deletion strain by RNAseq, using RNAseq data obtained from the 48-h timepoint of the N402 strain obtained from the same controlled conditions as a reference. 

**Figure 5:**
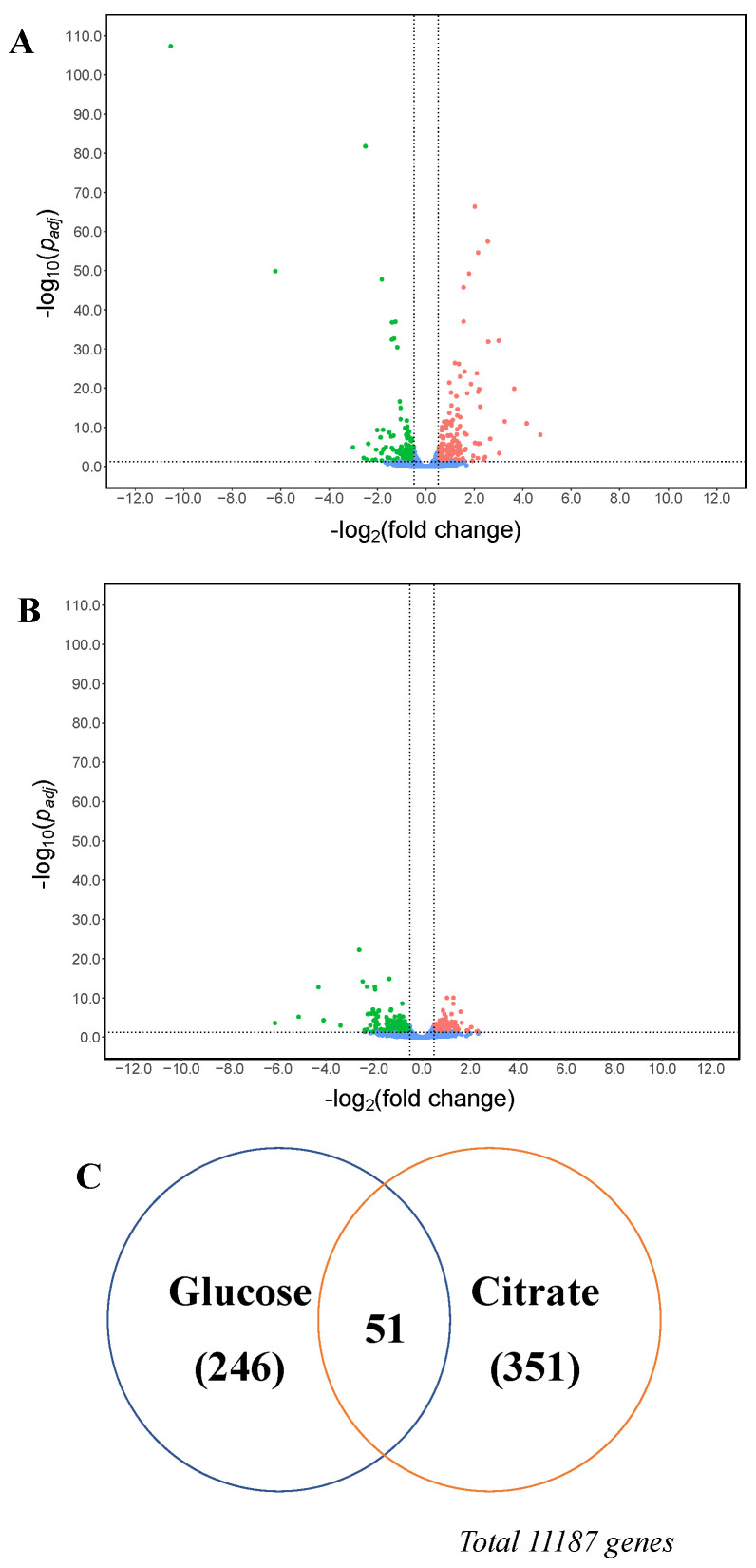
Volcano plot of gene transcript levels in terms of log_2_(fold change) of *A. niger ΔcitT* grown in glucose (**A**) and citrate (**B**) medium. Data of *A. niger* strain N402 was used as the reference. (**C**) Venn diagram of the *A. niger ΔcitT* genes expressed in glucose and citrate conditions. The regions between the dotted lines indicate the non-significant data.

When the transcriptional landscapes of both strains grown in glucose were compared, we observed that the deletion of the *citT* gene caused a significant change in the expression of 297 genes, 179 of them being up-regulated and 118 being down-regulated (Figure 5A and Supplementary Data S1 in sheet *ΔcitT*_vs_N402_glucose online). On the other hand, when both strains were using citrate as a carbon source, the transcript levels of 141 genes were significantly increased in the *ΔcitT* strain while the expression of 261 genes was significantly decreased (Figure 5B and Supplementary Data S1 in sheet *ΔcitT*_vs_N402_citrate online). Irrespective of the carbon source, as a consequence of the *citT* gene deletion, 27 genes were up-regulated (Table 2) and 23 genes were down-regulated (Figure 5C and Table 3). 

**Figure 6:**
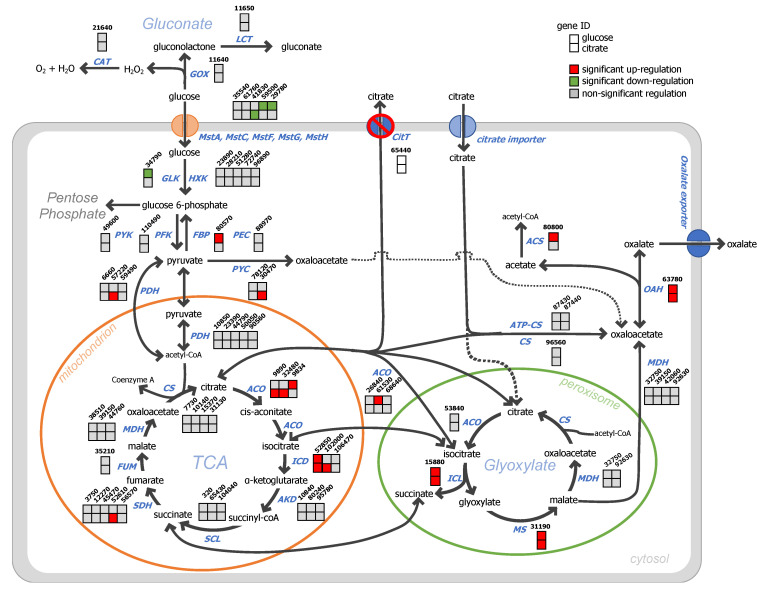
Expression of genes relating in organic acid pathways of *A. niger ΔcitT* grown in glucose (upper square) and citrate (lower square) medium. Data of *A. niger* strain N402 was used as reference. The solid arrows indicate the main routes of the pathway and the dotted arrows was alternative routes.

An overview of the main differentially expressed metabolic and transporter genes is provided (Figure 6). Since active citrate secretion is blocked in the glucose condition, it appeared that to eliminate intracellular citrate accumulation, two main strategies are used by the *ΔcitT* strain: (i) reduction of the glucose uptake rate and (ii) production of oxalic acid. At the molecular level, a reduction of glucose uptake was achieved by down-regulation of the transcript levels of the genes encoding the high-affinity glucose transporters *mstG* (ATCC64974_41830) and *mstH* (ATCC64974_29780) [16]. Because the tricarboxylic acid (TCA) cycle cannot act as a carbon sink, the carbon flux towards pyruvate decreased by down-regulation of the transcript level of the glucokinase encoding gene, an enzyme that phosphorylates glucose to glucose-6-phosphate and up-regulation of gene ATCC64974_80570 encoding fructose 1,6-biphosphatase normally involved in gluconeogenesis. 

To further reduce citrate accumulation, several genes facilitating oxalate production were significantly up-regulated in the *citT* deletant strain. In *A. niger*, oxalate is derived from oxaloacetate acetylhydrolase (OAH) [44], and the expression of the OAH encoding gene increased approximately four-fold. Alternatively, citrate can be converted to isocitrate by aconitase. In the *ΔcitT* strain, the transcript levels of the genes encoding a mitochondrial aconitase (ATCC64974_98340) and a cytosolic aconitase (ATCC64974_61530) were significantly increased. Additionally, the genes encoding enzymes of the glyoxylate cycle, isocitrate lyase and malate synthase were strongly up-regulated (Figure 6 and Supplementary Data S1 in sheet organic acid pathways online). 

Upon deletion of the *citT* gene the expression of 12 and 25 genes encoding proteins of the Major Facilitator Superfamily (MFS; PF07690) significantly changed under glucose and citrate conditions, respectively. In response to a knock-out of the citrate exporter, transcript levels of most of these genes showed a down-regulation (9 genes in glucose and 21 genes in citrate). 

**Figure 7:**
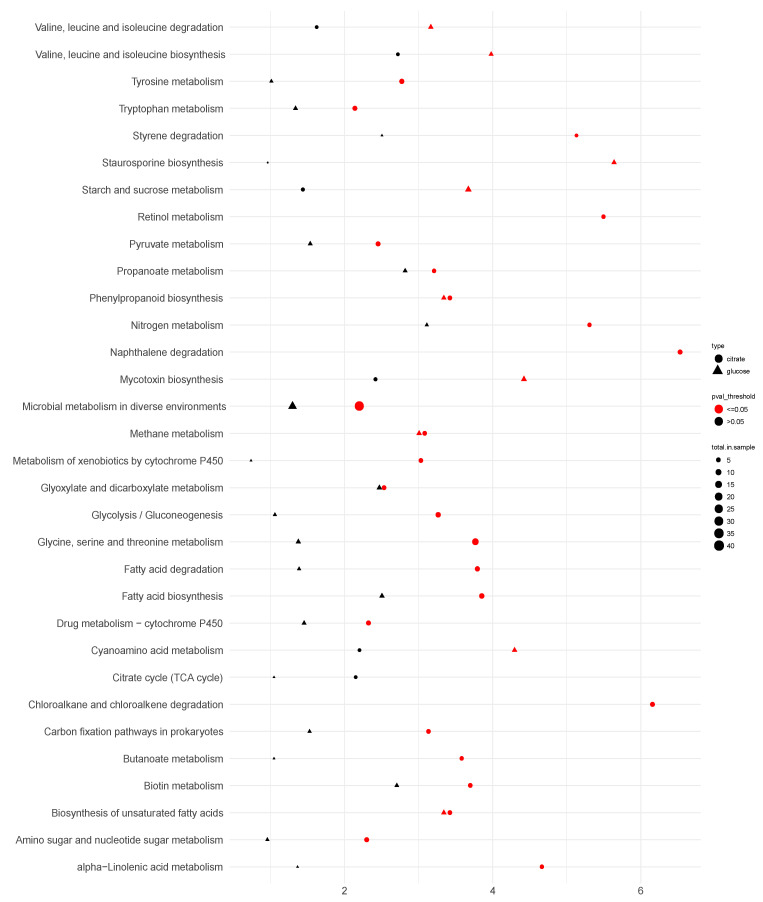
Scatter plot of pathway enrichment analysis of *A. niger ΔcitT* grown in glucose (▲) and citrate (⬤) medium. The *p*-value at ≤ 0.05 is represented in red and > 0.05 in black. Symbol size is related to the number of differentially expressed genes. Data of *A. niger* strain N402 was used as reference.

Overall, pathway enrichment analysis of the *ΔcitT* strain indicated that nine pathways were enriched in the glucose condition, whereas 25 pathways were shown to be enriched in the citrate condition (Figure 7 and Supplementary Data S1 in sheet pathway enrichment analysis online). Due to the deletion of the *citT* gene, three pathways showed enrichment irrespective of the carbon source. These three pathways were involved in (1) biosynthesis of unsaturated fatty acids, (2) methane metabolism and (3) phenylpropanoid biosynthesis. Although citrate cycle (TCA) was not significantly enriched in both carbon sources, significant enrichment of two pathways related to organic acids production, including glycolysis/gluconeogenesis and glyoxylate and dicarboxylate metabolism, was observed in citrate medium. 

### 3.6. Distribution of the Citrate Exporter Gene among Aspergillus Species

**Figure 8:**
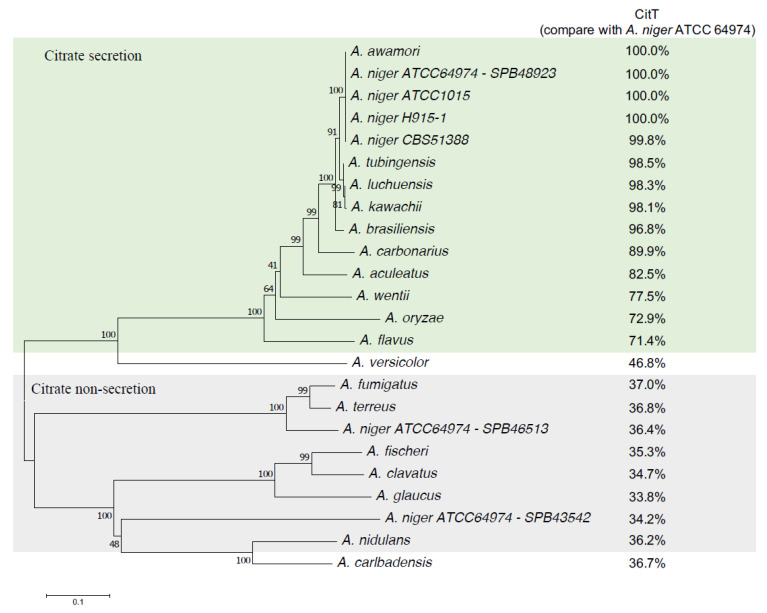
Phylogenetic relationships of the top-scoring candidate citrate exporters (CitT) of *Aspergillus* species. Known citrate secretors are shaded green, known citrate non-secretion are shaded gray. For *A. versicolor* and *A. carlbadensis* no data on citrate production could be found. *A. niger* SPB46513 and SPB43542 are best-hit paralogous sequences with homologs in non-secretors. Deduced amino acid sequences were aligned using ClustalX, followed by analysis using neighbor-joining in MEGA7. Numbers at nodes indicate bootstrap support following 1000 iterations. The protein sequences are found in Supplementary File S1 online.

*Aspergillus* section *Nigri* are prolific producers of in particular citric acids which suggests that a specialized exporter function has evolved for this purpose. The phylogenetic distribution of the *citT* gene among the various *Aspergillus* species was investigated by linking Blast similarity scores using the CitT protein as query with the reported ability to secrete citrate obtained from the literature (Figure 8). The analysis showed a clear separation into two groups. Although the query coverage for both groups was shown to be more than 90%, the percentage of sequence identity between both groups differed vastly. The sequence identity between the CitT protein and homologous proteins from other known citrate producing *Aspergillus* species was more than 71%, while the sequence identity between the *A. niger* citrate transporter and the best hit from non-producers was 42% or less. Using a bidirectional blast approach, proteins of the second group showed a better hit with two other *A. niger* N402 transporter proteins (Figure 8). Oxaloacetate hydrolase (OAH) is a member isocitrate lyase/PEP mutase enzyme superfamily of which multiple copies are present in *Aspergillus* species [45]. Taking advantage of the fact that a conserved active site serine is associated with oxaloacetate hydrolase (OAH) activity [46], the different species were investigated for the presence of the OAH encoding gene. All citrate secreting species also have an OAH encoding gene although the distribution among the citrate non-producers varies. 

## 4. Discussion

Next to establishing the most optimal fermentation conditions and unravelling the biochemistry of citric acid formation, at least three major regulatory steps of membrane transport, namely (1) hexose uptake, (2) citrate export from the mitochondrion to cytosol and (3) citrate export through the plasma membrane have been considered to be means to further improve citrate production in *A. niger* [13,47]. Limited research on citrate plasma membrane transport systems has been done [20,21,22,31]. Due to the significant difference in pH between the cytosol and the extracellular medium, citrate is present in a dissociated form (citrate^2−^) in the cytosol, at a pH between 6.0 and 7.0, and present in an undissociated form in the extracellular environment with a pH below 2.0 [8,9,10]. Both forms of citrate can cross the cell wall and export as well as uptake of citric acid in *A. niger* occurs by separate proton-dependent active symport systems. This transport is considered to be an industrial bottleneck for citrate production [48]. 

Recently, a gene facilitating citrate secretion (*citT*, alternative name *cexA*) of *A. niger* was discovered and partially characterized [19,20,21]. The RNAseq analysis we did in this study indicated that *citT* expression levels can vary at least a hundred-fold (Table 1). Furthermore, a sequence alignment between the single copy *citT* gene of N402 with the single copy *citT* gene from the heavy mutagenized industrial citrate-hyperproducing strain H915-1 indicated an almost hundred percent identity in nucleotide sequence of the gene and upstream and downstream region, which may suggest that nor the expression level of the exporter gene or exporter itself is a limiting factor in citrate production. 

### 4.1. citT Is Responsible for Active A. niger Citrate Export, but Not for Citrate Uptake

In this study, the consequences of deleting the *A. niger* citrate exporter gene, effectively blocking active citrate export, were examined by studying the consumption of glucose and citrate as carbon sources, monitoring the secretion of organic acids and by transcriptome pathway enrichment analysis. The *citT* knock-out almost completely abolished citrate export under otherwise citrate promoting conditions. (Table 1). However, we cannot exclude that other transporters have supportive roles in the secretion process. For instance, in the *citT* knock-out strain in both carbon sources, a down-regulation was observed for transporter gene ATCC64974_21300. ATCC64974_21300 is not functionally characterized but a BlastP analysis suggested that it could be a drug/H^+^ antiporter mediating the efflux of a variety of toxic compounds. Nevertheless, genome sequences of *Aspergillus* species known to secrete citrate clearly have homologs of the *citT* gene in single copy, while the second-best (endogenous) hit, encodes a different protein class with a significantly lower percentage of identity. Moreover, this protein class also present in some well-studied non-producers such as *A. fumigatus*. 

The *citT* (*cexA*) gene is reported as a gene controlling the intracellular and extracellular accumulation of citric acid in white and yellow koji fungi, *A. kawachii* and *A. oryzae,* respectively [49]. One homologous *cexA* gene was found in *A. kawachii* (*AkcexA*), which is a species phylogenetically related to *A. niger*, whereas *A. oryzae* carries two intrinsic *cexA* homolog (*AocexA* and *AocexB*). The results of both species showed in the same direction to *A. niger*. Disruption of *AkcexA* gene from *A. kawachii* genome led to significantly reduced citric acid accumulation, whereas overexpression of *AkcexA* into *A. oryzae* boosted both extracellular and intracellular citric acid accumulation in *A. oryzae* with a comparable level to *A. kawachii*. Additionally, the overexpression of either *AocexA* or *AocexB* into *A. oryzae* itself under a control of *amyB* promoter extremely enhanced the extracellular and intracellular citric acid accumulation [49]. The overexpression of *AkcexA* resulted in the up-regulation of 10 metabolites on the metabolic pathways closely linked to citric acid production including citric acid and isocitric acid (tricarboxylic acid (TCA) cycle); glucose 6-phosphate, and 3-phosphoglyceric acid (glycolysis); trehalose 6-phosphate (trehalose synthesis pathway); 6-phosphogluconic acid, ribose 5-phosphate, ADP-ribose, UDP, and NADP^+^ (pentose phosphate pathway) [49]. 

### 4.2. Blocking Active Citrate Export under Citrate Producing Conditions Reduces the Uptake of Glucose, Induces the Secretion of Oxalic Acid, and Activates the Glyoxylate Shunt

The transcriptome and pathway enrichment analysis in citrate exporting and importing conditions showed that in the presence of glucose, the high glycolytic flux normally present in citrate producing conditions was counterbalanced by a slower glucose consumption (Figure 4), coinciding at the molecular level with down-regulation of the genes encoding high-affinity glucose transporters, *mstG* (ATCC64974_41830) and *mstH* (ATCC64974_29780) (Figure 6 and Supplementary Data S1 in sheet organic acid pathways online). To further compensate for the absence of active citrate, transport oxalate production was significantly increased. Although it was reported that oxalate formation at the lower pH ranges from 1.5 to 2.5 is limited [9], in the fermentation experiment controlled at pH 2.5, oxalate was detected as the predominant organic acid at the maximum concentration that can be achieved under given conditions (Figure 4B). Transcriptome analysis provided molecular insight in how the surplus of citrate was converted to oxalate (Figure 6). First, citrate is converted by mitochondrial aconitases to *cis*-aconitate and then to isocitrate, respectively. The glyoxylate shunt is activated by the overexpression of the genes encoding isocitrate lyase and malate synthase. Malate is the main product of this pathways. It is used as a substrate to produce oxaloacetate by cytosolic malate dehydrogenase. Finally, oxaloacetate is hydrolyzed in oxalate and acetate by oxaloacetate acetylhydrolase (ATCC64974_63780). Upon oxalate secretion normally no accumulation of acetate is observed in the medium, which suggests that the acetate formed is quickly re-metabolized preventing secretion [6,9]. In the *ΔcitT* strain, up-regulation of acetyl-CoA synthase, an enzyme catalyzing the formation of cytosolic acetyl-CoA from acetate, was observed. (Figure 6 and Supplementary Data S1 in sheet organic acid pathways online). Using citrate as a substrate, normally ATP citrate lyase is responsible for the generation of cytosolic acetyl-CoA; however, we did not observe up-regulation of the two encoding genes. Pathway enrichment analysis showed that biosynthesis of unsaturated fatty acids (map01040) was significantly increased in the *citT* gene deletion strain. As a result, the knock-out might lead to production of fatty acids, especially linolenic acid, which was reported as a major unsaturated fatty acid produced under citrate accumulation conditions [50].

Citrate can be used as sole carbon and energy source by *A. niger* [48,51]. As the knock-out strain showed a normal growth on citrate this suggests that the fungus has a separate (active) citrate uptake system, as was first suggested by Netik et al. [48]. For this *A. niger* may have developed specific transporters facilitating citrate import or it may use carboxylic acid transporters, some of which have been shown to transport citrate [52] In the latter case ATCC64974_67850 and/or ATCC64974_45030, which are homologs of the genes encoding JenA and JenB carboxylic acid transporters in *A. nidulans*, respectively [53] are candidates, as both show high expression levels in citrate medium compared to glucose medium in both strains (Supplementary Data S1 in sheet All_RNASeq data online).

## 5. Conclusions

In summary, our study suggests that the *citT* gene is responsible for active *A. niger* citrate export, but not for citrate uptake. Blocking active citrate export under citrate producing conditions reduces the uptake of glucose, induces the secretion of oxalic acid and activates the glyoxylate shunt. Growth on citrate in addition leads to oxalic acid formation. Thus, by deleting a single gene, it is feasible not only to eliminate the undesired secondary formation of citric acid in the primary production of other organic acids such as oxalic and gluconic acid but also to significantly improve their production yields, even at low pH (pH 2.5). These insights are very valuable for the design and steering improved industrial production of organic acids by *A. niger*.

## Figures and Tables

**Table 1:** Transcript levels of genes in Major Facilitator Superfamily of *A. niger* N402 grown in citrate medium using glucose medium as the reference (the full list is in Supplementary Data S1 in sheet N402_MFS online).

Gene Locus Tag.	Protein ID	log_2_(Fold Change)	TMHs
ATCC64974_65440	SPB48923.1	−6.7765	12
ATCC64974_85340	SPB50921.1	−5.5734	12
ATCC64974_44020	SPB46772.1	−5.0525	10
ATCC64974_28550	SPB45214.1	−4.9393	12
ATCC64974_43890	SPB46759.1	−4.8217	8
ATCC64974_4840	SPB42837.1	−4.7665	12
ATCC64974_25910	SPB44949.1	−4.4900	12
ATCC64974_29710	SPB45330.1	−4.1990	11
ATCC64974_26160	SPB44974.1	−3.8357	12
ATCC64974_85360	SPB50923.1	−3.8232	11
ATCC64974_950	SPB42446.1	−3.7484	14
ATCC64974_26910	SPB45050.1	−3.5111	9
ATCC64974_93260	SPB51716.1	−3.2956	12
ATCC64974_72710	SPB49652.1	−3.2174	12
ATCC64974_21780	SPB44533.1	−3.1779	9
ATCC64974_105810	SPB52979.1	−3.1237	10
ATCC64974_72040	SPB49585.1	−3.0387	9
ATCC64974_10650	SPB43419.1	−3.0383	13

**Table 2:** Transcript levels of top up-regulated genes affected by the deletion of *citT* gene (Transcript levels are shown in log_2_(fold change) with the *p_adj_* ≤ 0.05).

	Protein ID	Description	log_2_(Fold Change)	
			Glucose	Citrate
ATCC64974_103440	SPB52738.1	2OG-Fe(II) oxygenase family oxidoreductase	4.7227	2.2847
ATCC64974_30940	SPB45454.1	carboxymuconolactone decarboxylase family protein	2.3894	1.6078
ATCC64974_6060	SPB42959.1	HMGL-like family protein	2.1506	1.1072
ATCC64974_91920	SPB51582.1	uncharacterized protein	2.1075	0.7707
ATCC64974_63780	SPB48756.1	oxaloacetate acetylhydrolase	2.0182	1.9265
ATCC64974_26450	SPB45003.1	zinc-binding dehydrogenase family protein	1.8572	1.4575
ATCC64974_15880	SPB43942.1	isocitrate lyase	1.5948	0.8524
ATCC64974_67790	SPB49158.1	acetyl-CoA hydrolase	1.5526	0.9054
ATCC64974_4720	SPB42825.1	O-methyltransferase family protein	1.4041	0.8684
ATCC64974_31190	SPB45479.1	malate synthase	1.3546	1.2960
ATCC64974_103400	SPB52734.1	uncharacterized protein	1.3405	1.3934
ATCC64974_18700	SPB44224.1	alpha/beta hydrolase family protein	1.2769	0.9495
ATCC64974_71600	SPB49541.1	uncharacterized protein	1.2265	0.8148
ATCC64974_4600	SPB42813.1	AMP-binding enzyme family protein	1.0504	0.6859
ATCC64974_58540	SPB48229.1	endo-polygalacturonase B	0.9209	0.7973
ATCC64974_63790	SPB48757.1	DUF1275 domain protein	0.9072	1.2557
ATCC64974_47060	SPB47077.1	methyltransferase domain family protein	0.7848	1.0456
ATCC64974_102280	SPB52622.1	glycosyl hydrolase family 61 family protein	0.7174	0.8594
ATCC64974_82680	SPB50654.1	WSC domain family protein	0.6728	0.8253
ATCC64974_4610	SPB42814.1	fungal Zn(2)-Cys(6) binuclear cluster domain family protein	0.6693	1.3609
ATCC64974_34830	SPB45846.1	TAP-like protein family protein	0.6533	0.7306
ATCC64974_104990	SPB52896.1	peptidase inhibitor I78 family protein	0.6393	0.5458
ATCC64974_64070	SPB48785.1	aldehyde dehydrogenase family protein	0.6174	0.7522
ATCC64974_63760	SPB48754.1	purine nucleoside permease	0.5466	0.9801
ATCC64974_34150	SPB45775.1	short chain dehydrogenase family protein	0.5283	0.6748
ATCC64974_52850	SPB47657.1	mitochondrial NADP- and NAD-isocitrate dehydrogenases	0.5169	0.6755
ATCC64974_34010	SPB45761.1	uncharacterized protein	0.5107	0.7421

**Table 3:** Transcript levels of top down-regulated genes affected by the deletion of *citT* gene (Transcript levels are shown in log_2_(fold change) with the *p_adj_* ≤ 0.05).

Gene Locus Tag	Protein ID	Description	log_2_(Fold Change)	
			Glucose	Citrate
ATCC64974_89450	SPB51334.1	uncharacterized protein	−6.2145	−4.0918
ATCC64974_92610	SPB51651.1	uncharacterized protein	−3.0166	−1.3542
ATCC64974_1740	SPB42525.1	alcohol dehydrogenase	−2.4351	−1.2566
ATCC64974_12500	SPB43604.1	laccase	−2.3820	−0.7627
ATCC64974_93830	SPB51773.1	uncharacterized protein	−2.1959	−2.0151
ATCC64974_89550	SPB51344.1	acid-stable alpha-amylase	−1.8176	−0.7229
ATCC64974_88150	SPB51204.1	fungal Zn(2)-Cys(6) binuclear cluster domain family protein	−1.7703	−2.6046
ATCC64974_1920	SPB42543.1	uncharacterized protein	−1.7534	−0.7516
ATCC64974_96990	SPB52093.1	high-affinity xylose transporter (XltA)	−1.6515	−1.4477
ATCC64974_87010	SPB51090.1	uncharacterized protein	−1.5061	−0.7842
ATCC64974_23000	SPB44655.1	proteinase aspergillopepsin II	−1.3996	−1.0743
ATCC64974_81710	SPB50557.1	extracellular alpha-glucosidase aglU	−1.3178	−0.9158
ATCC64974_48920	SPB47263.1	carboxypeptidase	−1.0439	−1.0691
ATCC64974_88220	SPB51211.1	mago binding family protein	−0.8482	−0.5610
ATCC64974_26630	SPB45021.1	uncharacterized protein	−0.8254	−1.2211
ATCC64974_103090	SPB52703.1	glycine zipper 2TM domain family protein	−0.7409	−0.9068
ATCC64974_88240	SPB51213.1	ubiquitin-protein ligase E3 component	−0.7202	−0.5844
ATCC64974_14700	SPB43824.1	alpha-glucosidase	−0.7012	−0.7846
ATCC64974_88190	SPB51208.1	cytochrome P450 alkane hydroxylase	−0.6886	−0.9308
ATCC64974_88170	SPB51206.1	adenylate cyclase AcyA	−0.6885	−0.6351
ATCC64974_21300	SPB44485.1	MFS transporter	−0.6525	−0.6241
ATCC64974_88210	SPB51210.1	protoporphyrinogen oxidase	−0.6309	−0.5470

## Data Availability

The raw sequencing data (DNA and RNA) have been submitted to the European Nucleotide Archive (ENA) and can be found under the accession number ERS3465414.

## Supplementary Materials

The following are available online at https://www.mdpi.com/article/10.3390/jof7060409/s1. Supplementary Data S1: Transcriptomic and Pathway Enrichment Analysis; Supplementary Figure S1: Scheme representing experimental steps for deletion of *citT* gene from the *A. niger* genome; Supplementary Figure S2: Sequence identity and synteny between the proposed genome assemblies of *Aspergillus niger* strains N402 and H915-1; Supplementary Figure S3: Growth of *A. niger* strain N402 and *ΔcitT* on MM plates; Supplementary Figure S4: Fumaric acid production by fermentation in glucose medium of *A. niger* strain N402 and *ΔcitT;* Supplementary Table S1: Cell dry weight of *A. niger* strain N402 and *ΔcitT* grown on various carbon sources by fermentation; Supplementary Table S2: Primers used in this study; Supplementary File S1: Amino acid sequences of candidate citrate exporter (CitT) of *Aspergillus* species.
