# Supplementary material for: Global Transcriptional Response of Aspergillus niger to Blocked Active Citrate Export through Deletion of the Exporter Gene
Source: J Fungi (Basel). 2021 May 23;7(6):409. doi: 10.3390/jof7060409 (PMC8224569; doi:10.3390/jof7060409)
Supplement: Supplementary file 1 [file jof-07-00409-s001.zip › Supplementary Figure S1_Scheme of knockout.pdf]

Global transcriptional response of *Aspergillus niger* to blocked active citrate export through deletion of the exporter gene

Thanaporn Laothanachareon[1,2,a,*], Lyon Bruinsma[1], Bart Nijsse[1], Tom Schonewille[1], Maria Suarez Diez[1], Juan Antonio Tamayo-Ramos[3], Vitor AP Martins dos Santos[1,4,*#], Peter J. Schaap[1#]

[1] Laboratory of Systems and Synthetic Biology, Wageningen University & Research, Wageningen, The Netherlands

[2] Enzyme Technology Laboratory, Biorefinery and Bioproduct Research Group, National Center for Genetic Engineering and Biotechnology, 113 Thailand Science Park, Khlong Luang, Pathumthani 12120, Thailand

[3] International Research Center in Critical Raw Materials-ICCRAM, University of Burgos, Burgos, Spain.

[4]LifeGlimmer GmbH, Berlin, Germany

# Joint senior authors

*** Corresponding author:**

Thanaporn Laothanachareon

Mailing address: Enzyme Technology Laboratory, Biorefinery and Bioproduct Research Group, National Center for Genetic Engineering and Biotechnology, 113 Thailand Science Park, Khlong Luang, Pathumthani 12120, Thailand

Email: thanaporn.lao@biotec.or.th (TL)

Vitor AP Martins dos Santos

Mailing address: Laboratory of Systems and Synthetic Biology, Wageningen University & Research, Wageningen, The Netherlands

Email: vitor.martinsdossantos@wur.nl (VAPMS)

[a]Current address: Enzyme Technology Laboratory, Biorefinery and Bioproduct Technology Research Group, National Center for Genetic Engineering and Biotechnology, 113 Thailand Science Park, Khlong Luang, Patumthani 12120, Thailand

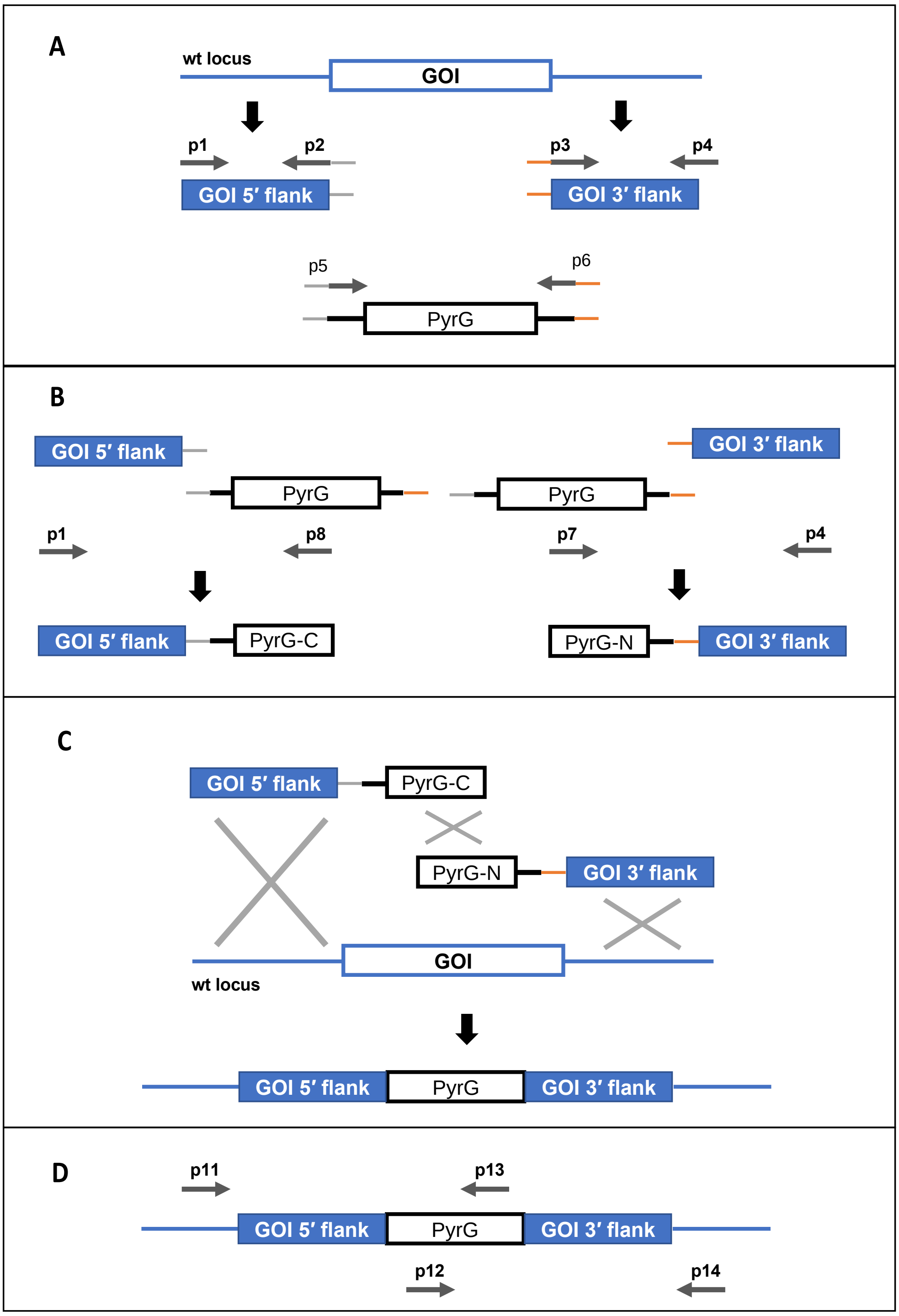

Supplementary Figure S1 | Scheme representing experimental steps for deletion of *citT* gene from the *A. niger* genome. A) The 5' flanking region of the *citT* gene was amplified with

primers citT_KO_5Flank_FW_p1 and citT_KO_5Flank_RV_p2. The 3' flanking region of the *citT* gene was amplified with primers citT_KO_3Flank_FW_p3 and citT_KO_3Flank_RV_p4. The *pyrG* gene was amplified with primers AOpyrG12FW_p5 and AOpyrG13RV_p6. B) Deletion of the *citT* gene by the split marker method consisting of two overlapping DNA fragments to disrupt of the *citT* gene. Fragment one contains the 5' flank of the *citT* gene and a partial version of the *pyrG* gene and was constructed by primers citT_KO_5Flank_FW_p1 and AOpyrG15RV_p8. Fragment two contains an overlapping partial version of the selection marker and the 3' flank of the *citT* gene and was established by using primers AOpyrG14FW_p7 and citT_KO_3Flank_RV_p4. C) Deletion of the *citT* gene by the split marker method using the two overlapping DNA fragments and transformation in *A. niger* strain MA169.4. D) Confirmation of the integration position. The citT_KO_FW_p11 and AOpyrG_KO_RV_p13 and the AOpyrG_KO_FW_p14 and citT_KO_RV_p12 were applied to check on the 5′flanking and the 3′ flanking region, respectively. The gene replacement and the purity of the knock-out strains were also checked (scheme does not show). The AOpyrG13RV_p6 and AOpyrG13RV_p6 primers were used to determine the *pyrG* marker gene replacement and the citT_1FW and citT_1770RV primers were verified the purity of the knock-out strains.
