# Supplementary material for: Global Transcriptional Response of Aspergillus niger to Blocked Active Citrate Export through Deletion of the Exporter Gene
Source: J Fungi (Basel). 2021 May 23;7(6):409. doi: 10.3390/jof7060409 (PMC8224569; doi:10.3390/jof7060409)
Supplement: Supplementary file 1 [file jof-07-00409-s001.zip › Supplementary Figure S2_Circos plot.pdf]

Global transcriptional response of *Aspergillus niger* to blocked active citrate export through deletion of the exporter gene

Thanaporn Laothanachareon[1,2,a,*], Lyon Bruinsma[1], Bart Nijsse[1], Tom Schonewille[1], Maria Suarez Diez[1], Juan Antonio Tamayo-Ramos[3], Vitor AP Martins dos Santos[1,4,*#], Peter J. Schaap[1#]

[1] Laboratory of Systems and Synthetic Biology, Wageningen University & Research, Wageningen, The Netherlands

[2] Enzyme Technology Laboratory, Biorefinery and Bioproduct Research Group, National Center for Genetic Engineering and Biotechnology, 113 Thailand Science Park, Khlong Luang, Pathumthani 12120, Thailand

[3] International Research Center in Critical Raw Materials-ICCRAM, University of Burgos, Burgos, Spain.

[4]LifeGlimmer GmbH, Berlin, Germany

# Joint senior authors

*** Corresponding author:**

Thanaporn Laothanachareon

Mailing address: Enzyme Technology Laboratory, Biorefinery and Bioproduct Research Group, National Center for Genetic Engineering and Biotechnology, 113 Thailand Science Park, Khlong Luang, Pathumthani 12120, Thailand

Email: thanaporn.lao@biotec.or.th (TL)

Vitor AP Martins dos Santos

Mailing address: Laboratory of Systems and Synthetic Biology, Wageningen University & Research, Wageningen, The Netherlands

Email: vitor.martinsdossantos@wur.nl (VAPMS)

[a]Current address: Enzyme Technology Laboratory, Biorefinery and Bioproduct Technology Research Group, National Center for Genetic Engineering and Biotechnology, 113 Thailand Science Park, Khlong Luang, Patumthani 12120, Thailand

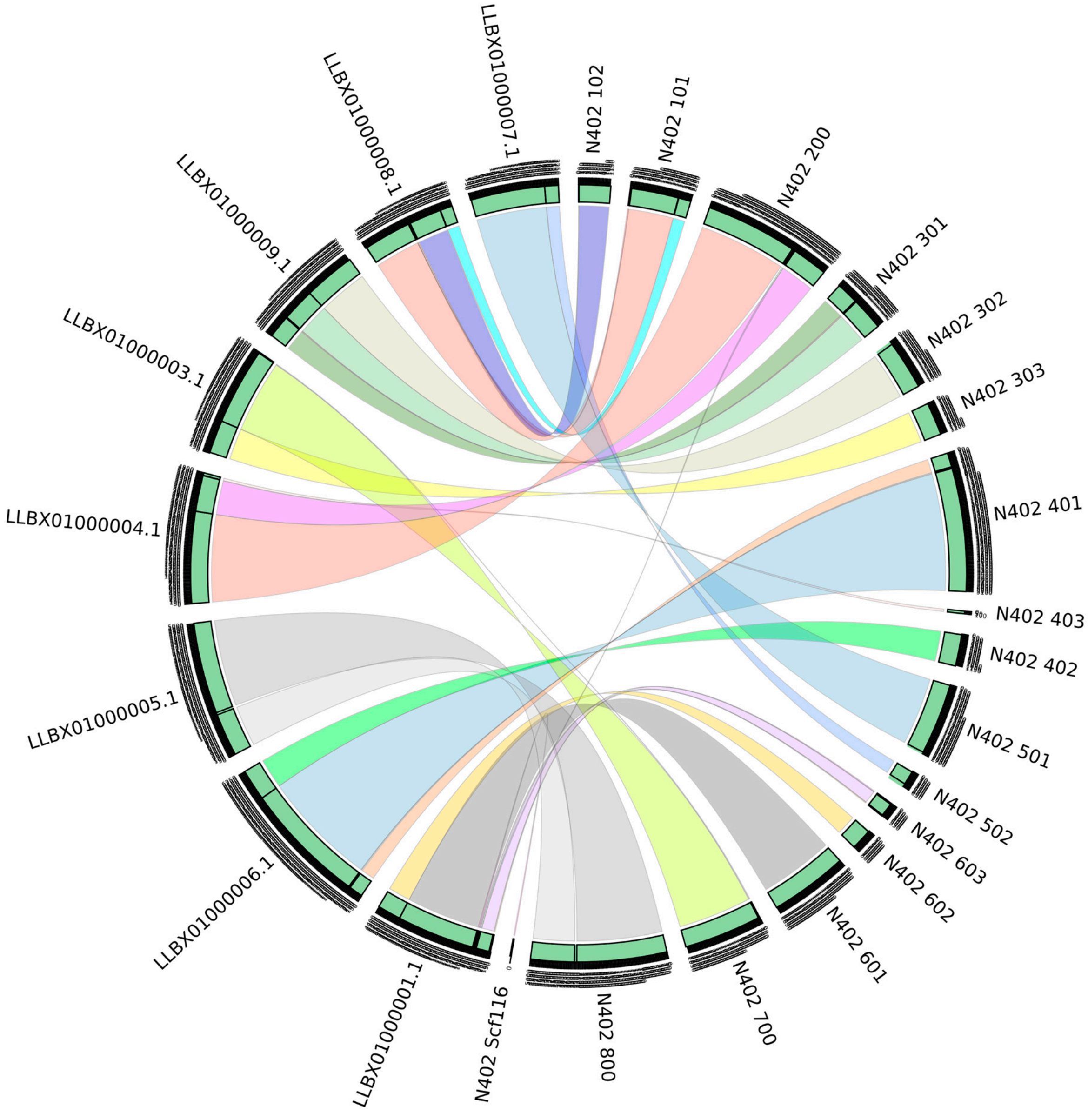

Supplementary Figure S2 | Sequence identity and synteny between the proposed genome assemblies of *Aspergillus niger* strains N402 and H915-1. The genome comparison is plotted in circos format (Krzywinski et al., 2009).

Krzywinski, M. et al. Circos: an information aesthetic for comparative genomics. Genome research 19, 1639-1645, doi:10.1101/gr.092759.109 (2009).
