# Supplementary material for: Global Transcriptional Response of Aspergillus niger to Blocked Active Citrate Export through Deletion of the Exporter Gene
Source: J Fungi (Basel). 2021 May 23;7(6):409. doi: 10.3390/jof7060409 (PMC8224569; doi:10.3390/jof7060409)
Supplement: Supplementary file 1 [file jof-07-00409-s001.zip › Supplementary Figure S3_plates.pdf]

Global transcriptional response of *Aspergillus niger* to blocked active citrate export through deletion of the exporter gene

Thanaporn Laothanachareon[1,2,a,*], Lyon Bruinsma[1], Bart Nijsse[1], Tom Schonewille[1], Maria Suarez Diez[1], Juan Antonio Tamayo-Ramos[3], Vitor AP Martins dos Santos[1,4,*#], Peter J. Schaap[1#]

[1] Laboratory of Systems and Synthetic Biology, Wageningen University & Research, Wageningen, The Netherlands

[2] Enzyme Technology Laboratory, Biorefinery and Bioproduct Research Group, National Center for Genetic Engineering and Biotechnology, 113 Thailand Science Park, Khlong Luang, Pathumthani 12120, Thailand

[3] International Research Center in Critical Raw Materials-ICCRAM, University of Burgos, Burgos, Spain.

[4]LifeGlimmer GmbH, Berlin, Germany

# Joint senior authors

*** Corresponding author:**

Thanaporn Laothanachareon

Mailing address: Enzyme Technology Laboratory, Biorefinery and Bioproduct Research Group, National Center for Genetic Engineering and Biotechnology, 113 Thailand Science Park, Khlong Luang, Pathumthani 12120, Thailand

Email: thanaporn.lao@biotec.or.th (TL)

Vitor AP Martins dos Santos

Mailing address: Laboratory of Systems and Synthetic Biology, Wageningen University & Research, Wageningen, The Netherlands

Email: vitor.martinsdossantos@wur.nl (VAPMS)

[a]Current address: Enzyme Technology Laboratory, Biorefinery and Bioproduct Technology Research Group, National Center for Genetic Engineering and Biotechnology, 113 Thailand Science Park, Khlong Luang, Patumthani 12120, Thailand

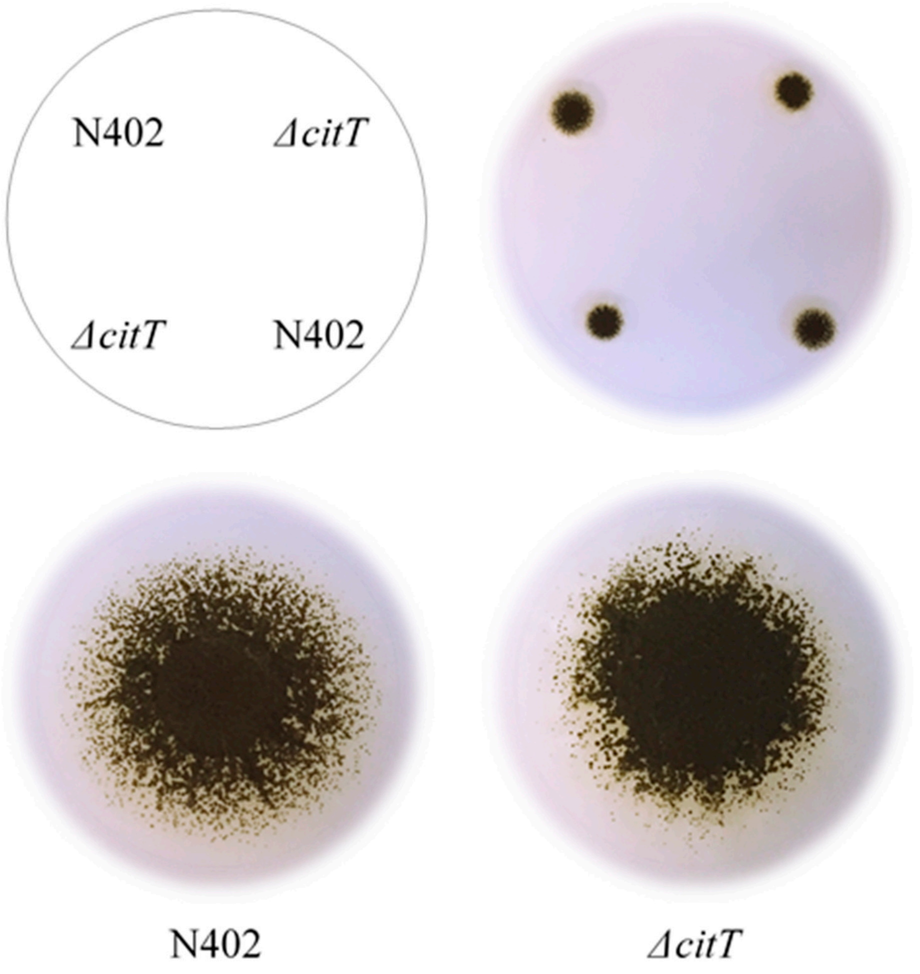

Supplementary Figure 3 | Growth of *A. niger* strain N402 and *ΔcitT* on MM plates (20 mL/L 50% glucose, 20 mL/L 50x ASPA + N, 2 mL/L 1 M $MgSO_4$, 1 mL/L 1000x Vishniac and 15 g/L agar) at 30°C for 3 days.
