# Supplementary material for: Global Transcriptional Response of Aspergillus niger to Blocked Active Citrate Export through Deletion of the Exporter Gene
Source: J Fungi (Basel). 2021 May 23;7(6):409. doi: 10.3390/jof7060409 (PMC8224569; doi:10.3390/jof7060409)
Supplement: Supplementary file 1 [file jof-07-00409-s001.zip › Supplementary File S1_CitT sequences.pdf]

Global transcriptional response of *Aspergillus niger* to blocked active citrate export through deletion of the exporter gene

Thanaporn Laothanachareon[1,2,a,*], Lyon Bruinsma[1], Bart Nijsse[1], Tom Schonewille[1], Maria Suarez Diez[1], Juan Antonio Tamayo-Ramos[3], Vitor AP Martins dos Santos[1,4,*#], Peter J. Schaap[1#]

[1] Laboratory of Systems and Synthetic Biology, Wageningen University & Research, Wageningen, The Netherlands

[2] Enzyme Technology Laboratory, Biorefinery and Bioproduct Research Group, National Center for Genetic Engineering and Biotechnology, 113 Thailand Science Park, Khlong Luang, Pathumthani 12120, Thailand

[3] International Research Center in Critical Raw Materials-ICCRAM, University of Burgos, Burgos, Spain.

[4]LifeGlimmer GmbH, Berlin, Germany

# Joint senior authors

*** Corresponding author:**

Thanaporn Laothanachareon

Mailing address: Enzyme Technology Laboratory, Biorefinery and Bioproduct Research Group, National Center for Genetic Engineering and Biotechnology, 113 Thailand Science Park, Khlong Luang, Pathumthani 12120, Thailand

Email: thanaporn.lao@biotec.or.th (TL)

Vitor AP Martins dos Santos

Mailing address: Laboratory of Systems and Synthetic Biology, Wageningen University & Research, Wageningen, The Netherlands

Email: vitor.martinsdossantos@wur.nl (VAPMS)

[a]Current address: Enzyme Technology Laboratory, Biorefinery and Bioproduct Technology Research Group, National Center for Genetic Engineering and Biotechnology, 113 Thailand Science Park, Khlong Luang, Patumthani 12120, Thailand

Supplementary File S1 | Amino acid sequences of candidate citrate exporter (CitT) of *Aspergillus* species

```
>A. awamori
MSSTTSSSRSDLEKVPVPQVTPRDSDSDKGSLSPEPSTLEAQSSEKPPHHIFTRSRKLQMVCIVSLAAIFSPLSS
NIYFPALDDVSKSLNISMSLATLTITVYMIVQGLAPSFWGSMSDATGRRPVFIGTFIVYLVANIALAESKNYGEL
MAFRALQAAGSAATISIGAGVIGDITNSEERGSLVGIFGGVRMLGQGIGPVFGGIFTQYLGYRSIFWFLTIAGGV
SLLSILVLLPETLRPIAGNGTVKLNGIHKPFIYTITGQTGVVEGAQPEAKKTKTSWKSVFAPLTFLVEKDVFITL
FFGSIVYTVWSMVTSSTTDLFSEVYGLSSLDIGLTFLGNGFGCMSGSYLVGYLMDYNHRLTEREYCEKHGYPAGT
RVNLKSHPDFPIEVARMRNTWWVIAIFIVTVALYGVSLRTHLAVPIILQYFIAFCSTGLFTINSALVIDLYPGAS
ASATAVNNLMRCLLGAGGVAIVQPILDALKPDYTFLLLAGITLVMTPLLYVEDRWGPGWRHARERRLKAKANGN

>A. aculeatus
MSLSREETAKVPEPQLSSVASFTDQDSEKGSISRDPSTFEGQSSEPPPHHIFTRSRKLQMVCIVSMAAIFSPLSS
NIYFPALDDVSKSLNVSMTLATLTITIYMIVQGLAPTFWGSLSDALGRRPVFIGTFVVYLVANIALAESKNYGEL
MAFRALQAAGSAATISIGKIVIGIHCCSTPNAYAGAGVIGDITTSAERGSLVGIFGGVRMLGQGIGPVFGGLLTQ
YLGYRSIFWFLTILGGVSLVSILVLLPETLRPIAGNGTVKLDGLHKPFLYHFTSQPGVIDGAEPGVKKAKVTWRT
VCAPLKFLAEKDVFITLFFGSIVYTVWSMVTSSTTDLFSEVYGLSSLEVGLTFLGNGFGCMSGSYLVGYLMDYNH
RLTEREYCEKHGYPAGTRVNLKSHSDFPIEVARMRNTWWITGIFIVTTAVYGVSLRTHVAVPIILQYFIAFCATG
IFTINSALVIDLYPGASASATAVNNLMRCLIGAAGVAAVQPILEALGADYTFVLLAGITLVMVPLLWIEDRWGPG
WRHARERRLKAQMP

>A. brasiliensis
MSSTTSSSRSDLEKVPVTQVTPRDNDSDKGSVSPEPSTLEAQSSEKPPHHIFTRSRKLQMVCIVSLAAIFSPLSS
NIYFPALDDVSKSLNISMSLATLTITVYMIVQGLAPSFWGSLSDATGRRPVFIGTFVVYLVANIALAVSKNYGEL
MAFRALQAAGSAATISIGAGVIGDITNSEERGSLVGIFGGVRMLGQGIGPVFGGIFTQYLGYRSIFWFLTIFGGV
SLLSILVLLPETLRPIAGNGSVKLHGIHKPFIYMITGQKGVIEGAQPEAKKTKTSWKSVFAPLTFLIEKDVFITL
FFGSIVYTVWSMVTSSTTDLFSEVYGLSSLDIGLTFLGNGFGCMSGSYLVGYLMDYNHRLTEREYCEKHGYPAGT
RVNLKSHPDFPIEVARMRNTWWVIAIFIVTVAVYGVSLRTHLAVPIILQYFIAFCSTGLFTINSALVIDLYPGAS
ASATAVNNLMRCLLGAGGVAVVQPILDALNPDYTFLLLAGITLVMTPLLYVEDRWGPGWRHARERRLKAKANSN

>A. carbonarius
MSSRTDIEKVPEPQVTPKDLDSEKDATSPEPSVLESQPSEKPPHHIFTRSRKLQMVGIVSLAAIFSPLSSNIYFP
ALDDVSQSLHISMSLATLTITVYMIVQGLAPSFWGSLSDATGRRPVFIGTFIVYLIANIALGESKNYGELMAFRA
```

LQAAGSAATISIGAGVIGDITTSEERGSLVGIFGGVRMLGQGIGPVFGGIFTQYFGYRSIFWFLTAFGGVSLLSI
MVLLPETLRPIAGNGTVKLTGMHKPFIYSITGQKGVIEGALPDARKGKVTWRAVFAPLTFLIEKDVFITLFFGSV
VYTVWSMVTSSTTDLFSEVYGLSSLQIGLTFLGNGFGCMSGSYLVGYLMDYNHRLTEREYCEKHGYPAGTRVNLK
SHSDFPIEVARMRNTWWVIAIFVVATALYGVSLRTHLAVPIILQYFIAFCSTGLFTINSALVIDLYPGASASATA
VNNLMRCLLGAGGVAVVQLIIDALTAEYTFILLAGITLAMTPLLYIEDRWGPSWRHARERRLKAKANGN

>A. carlbadensis
MASTVEDKETAAAEQDTLRVAPYSAFSRAQKRFIVFSASWAGFFSPVSSQIYFPALNSVANDLGVSGALINLTLT
SYMIFQGLSPMFVGDFADKAGRRPAYLVCFIIYTAANIGLALQDNYAALFVLRCLQSAGSSTTIALSSGVVADIA
IAAERGSYMGFVTAGSLLGPALGPVVGGLLSQYLGWRSIFWFLVIFGCAFLVPFVLFFPETSRVIVGDGSIPAPP
WNQPLVQWLKTRKSPQQETITRPKLRFPNPLKTLSIVFQKDVSIILLANAVLFAGFYDVSASIPSIFEDLYGLDD
LQIGLCYIPFGLGATVAALITGKMLDRNFRRLARLSNIPLEETRARNLQGFPIEKARLQVALPTLLMGSSTVIAF
GWVLHFGVHIAAPTIILFFMGLTLTGAFNTVSTLLVDFYPMNAASATASNNFVRCLLGAGATALIDPMLRAMGRG
WCFTFIALVMMSTTPLLLVIMHFGPRWREERQRKHEAR

>A. clavatus
MSTGVREVQEGRAAASSSSNDGDNVIQYHHVDRPAEAASGSDPSALEAQKETTLQVDTEQAHSAFTQNEKRFIIL
MISMASFFSPLSGQIYFPVMPTLVKNYHLTTALINLTITTYMILQGLAPSFMGTFADSGGRRPAYILAFAIYTAA
NIGLALQNSYVALMILRCVQSAGSSGTVAFGYGVVSDIATAAERGSYIGPMAAGVMVAPAIGPVIGGILAKFLGW
RSVFWFLVIISGGYLVVFTIAVPETARTIVGDGSVPPKESWRMSVVQYWSARRRLRKMSVEERRVHEEQRARLNQ
QHGKKKIAFPNPLEAFAILLEKDALIIISYVGLAMLSNTALLTSIPNLFGKLYGFNDLQIGLCFLPLGVSSCIAA
VLNGKLLDFNYRRTARKLGLSVDKKKGDDLRGFPIEQTRLQTFFPIMAVGVAAFIPYGWVLQQRTPLVAPLILQF
IIGFSFIASLNTLNTLMVDLFPDRAATASAASNLVRCWLGAVGAAVVDHMLSAMGWGWCFAFWGLLMLAGFALLL
LEYRHGMKWRLARLTKLDQIKAAKQAEKEAQQAADETNEDKKDAYGPNSGPNDGSAK

>A. flavus
MGQPNDLKSPEVASVPVSSKNSISDPSSPLEDSLEAQQPSPPIYHIFSRSQKLEMVVIVSLAAIFSPLSSNIYFP
ALGAISRDLHTSMTLTTLTVTIYMIVQGIAPTFWGSISDTSGRRPVFIGTMVVYIIANIALAVSTNYGELMAFRA
LQAAGSAATISIGAGVIGDVTTSAERGSLLTVRQVRMLGQGIGPVFGGLLAQYLGFRSIFWFLTICASVSLFTIL
LLLPETLRSIAGNGTVPLRGLQKPWLYYITGQPGAEEGAESGIKKSRVTFGTVFAPLKFLFEIDVFITLFFGSIV
YTVWSMVTSSTSDLFEETYNLTTLQVGLTFLGNGSYTIGYLMDYNHRLTEREYCEKHNYPPGTRVNLKTHPDFPI
ETARMRNTWWITVIFIVCVAVYGVSLRTHLAVPIILQYIIAYCSTGIFTINSALVIDLYPGASASATAVNNLMRC
LIGAAGVAAVQPIIDALGPTYTFVLLAGITLVLCPLLWVETNYGAGWRLARHQRLNRPRAG

>A. fumigatus
MSSTGGKTPTSNTKTHPTVMTDPPYSIFDTRQKWLIIIIVSTAATFSGFASNIYFPALPTIAIDLNVSLELVNLT
VTSYLIFQGLAPSFWGPVSDVKGRRTAYICTFIVFFCACIGLAETKNYTTLIVVRCLQSAGSASTIAIGSGVIGD
ITTRADRGGYMGVFQAGLLVPVAVGPVIGGAIAGSLGWKAIFWFLAIYSGVFLCLLTLVLPETLRSIVGNGSRKP
SHPVLRYPLNLYQKSSKMPWQQPQDGLSTAEAKKKIDLLGPLRMLLSNHAAPIILFLAVYYAVWQMSITAMSSLF
KSRYGLSELQIGLTFIANGVGSMVGTLVTGKILDADYRRVKTKYEASFDNEHRDAMSQTAREENFPLESARLRLV

PIFSITQCVSIILFGWTIQYPDKVHIAVPIVSTFITGWTAVSTQSLIMTYLVDLFPDRSAAASASLNLARCLFAA
GGTSFIMPMINGVGVGVAFTICVAVQMVALIGPLIQWRFAAGWRRKEREEAAKREGQGEC

>A. glaucus
MENERRKESDSTMASLSTSHTVKDTTPDIVECKTDGGSSQDHRGSENDGKCDCEQADKEPSTLEAQPADEQTPVH
SAFTKNEKRFIISMISMASFFSPLSGQIYYPVMPTLVRNYHLTTALVNLTVTTYMILQGLAPSFMGTFADSGGRR
PAYILAFVIYTAANIGLAVQDSFAALLVLRCLQSAGSSGTVSFGYGVVSDIATPSERGRFVGPMAAGVMVAPALG
PVIGGILAKFLGWRSVFWFLVIISGGYLVVFAIMMPETARRVVGNGSVPPKEWWRMSLIQYLAERRRVKKMSAEE
REGHEEQQLALSNNASHTRKLKFPNPLETFAILLEKDALIIITFIGIVMFANIALLTSTPNIFTKLYGFNDLQIG
LCFLPLGTSACLAAILNGRLLDWNYRRTATRLGFHIDRKKGDDIRTFPIEKTRLQTFFPLMSVGILTYLPYGWVL
QKRAPLVAPLILQFIIGFCFVAALNTLNTLIVDLFPDRSATAAAANNLVRCWLGAVGAALIDQMLRGMGGGWCFT
FLGLVMAVGLGFVSLEGKYGMEWREQRRVKMEKKKEKKERKEEKKQEEQIKG

>A.kawachii
MSSTTSSSRSDLEKVPVPQVTPRDNDSDKGSLSPEPSTLEAQSSEKPPHHIFTRSRKLQMVCIVSLAAIFSPLSS
NIYFPALDDVSKSLNISMSLATLTITVYMIVQGLAPSFWGSMSDATGRRPVFIGTFVVYLVANIALAESKNYGEL
MAFRALQAAGSAATISIGAGVIGDITNSEERGSLVGIFGGVRMLGQGIGPVFGGIFTQYLGYRSIFWFLIIAGGV
SLLSILVLLPETLRPIAGNGTVKLNGIHKPFIYSITGQKGVVEGAQPEAKKKQSSWKSVFAPLTFLVEKDVFITL
FFGSIVYTVWSMVTSSTTDLFSEVYGLSSLDIGLTFLGNGFGCMSGSYLVGYLMDYNHRLTEREYCEKHGYPAGT
RVNLKSHPDFPIEVARMRNTWWVIAIFIVTVALYGVSLRTHLAVPIILQYFIAFCSTGLFTINSALVIDLYPGAS
ASATAVNNLMRCLLGAGGVAVVQPILDALNPDYTFLLLAGITLVMTPLLYVEDRWGPGWRHARERRLKAKANGN

>A. luchuensis
MSSTTSSSRSDLEKVPVPQVTPRDNDSDKGSLSPEPSTLEAQSSEKPPHHIFTRSRKLQMVCIVSLAAIFSPLSS
NIYFPALDDVSKSLNISMSLATLTITVYMIVQGLAPSFWGSMSDATGRRPVFIGTFVVYLVANIALAESKNYGEL
MAFRALQAAGSAATISIGAGVIGDITNSEERGSLVGIFGGVRMLGQGIGPVFGGIFTQYLGYRSIFWFLTIAGGV
SLLSILVLLPETLRPIAGNGTVKLNGIHKPFIYSITGQKGVVEGAQPEAKKKQSSWKSVFAPLTFLVEKDVFITL
FFGSIVYTVWSMVTSSTTDLFSEVYGLSSLDIGLTFLGNGFGCMSGSYLVGYLMDYNHRLTEREYCEKHGYPAGT
RVNLKSHPDFPIEVARMRNTWWVIAIFIVTVALYGVSLRTHLAVPIILQYFIAFCSTGLFTINSALVIDLYPGAS
ASATAVNNLMRCLLGAGGVAVVQPILDALNPDYTFLLLAGITLVMTPLLYVEDRWGPGWRHARERRLKAKANGN

>A. nidulans
MASPQTPSKEGDTGLKGGLDVVATNTTGEVPYSVFTKAQKRYIVFFASWAGFFSPVSSQIYFPALNSIADDLGVT
SALINLTLTSYMIFQGVSPMFVGDFADKAGRRPAYMVCFLIYIAANIGLALQDNFAALFVLRCLQSAGSSTTIAL
SAGVVSDIAIAAERGSYMGFVTAGSLLGPAMGPVIGGLLSQYLGWRSVFWFLTIFAGAFLVPFVLLFPETARAIV
GNGSIPPPKWNIPLLTLFQARKEPQPEDFDWPKLRFPNPIRTLSIVFQKDIAIILIANAILFAGFYDVTAAIPSI
YNELYGLDDLYIGLCYVPFGLGATVASIATGKLLDFNYRRLAKQLNVPLQETRARNLTHFPIEFARLQVALPLLT
LGAFTIIAFGWCLNYGVHLAAPTTILFLMGLTLTGAFNTVSTLLVDFYPSNASAATASNNLVRCLLGAGATALID
PMLEAMGRGWCFTFIALVMLCTMPLLCVVMRWGPRWRNERQAKIDAATTGNCQN

>A. niger_ATCC1015
MSSTTSSSRSDLEKVPVPQVTPRDSDSDKGSLSPEPSTLEAQSSEKPPHHIFTRSRKLQMVCIVSLAAIFSPLSS
NIYFPALDDVSKSLNISMSLATLTITVYMIVQGLAPSFWGSMSDATGRRPVFIGTFIVYLVANIALAESKNYGEL
MAFRALQAAGSAATISIGAGVIGDITNSEERGSLVGIFGGVRMLGQGIGPVFGGIFTQYLGYRSIFWFLTIAGGV
SLLSILVLLPETLRPIAGNGTVKLNGIHKPFIYTITGQTGVVEGAQPEAKKTKTSWKSVFAPLTFLVEKDVFITL
FFGSIVYTVWSMVTSSTTDLFSEVYGLSSLDIGLTFLGNGFGCMSGSYLVGYLMDYNHRLTEREYCEKHGYPAGT
RVNLKSHPDFPIEVARMRNTWWVIAIFIVTVALYGVSLRTHLAVPIILQYFIAFCSTGLFTINSALVIDLYPGAS
ASATAVNNLMRCLLGAGGVAIVQPILDALKPDYTFLLLAGITLVMTPLLYVEDRWGPGWRHARERRLKAKANGN

>A. niger_ATCC64974_SPB48923
MSSTTSSSRSDLEKVPVPQVTPRDSDSDKGSLSPEPSTLEAQSSEKPPHHIFTRSRKLQMVCIVSLAAIFSPLSS
NIYFPALDDVSKSLNISMSLATLTITVYMIVQGLAPSFWGSMSDATGRRPVFIGTFIVYLVANIALAESKNYGEL
MAFRALQAAGSAATISIGAGVIGDITNSEERGSLVGIFGGVRMLGQGIGPVFGGIFTQYLGYRSIFWFLTIAGGV
SLLSILVLLPETLRPIAGNGTVKLNGIHKPFIYTITGQTGVVEGAQPEAKKTKTSWKSVFAPLTFLVEKDVFITL
FFGSIVYTVWSMVTSSTTDLFSEVYGLSSLDIGLTFLGNGFGCMSGSYLVGYLMDYNHRLTEREYCEKHGYPAGT
RVNLKSHPDFPIEVARMRNTWWVIAIFIVTVALYGVSLRTHLAVPIILQYFIAFCSTGLFTINSALVIDLYPGAS
ASATAVNNLMRCLLGAGGVAIVQPILDALKPDYTFLLLAGITLVMTPLLYVEDRWGPGWRHARERRLKAKANGN

>A. niger_CBS51388
MSSTTSSSRSDLEKVPVPQVIPRDSDSDKGSLSPEPSTLEAQSSEKPPHHIFTRSRKLQMVCIVSLAAIFSPLSS
NIYFPALDDVSKSLNISMSLATLTITVYMIVQGLAPSFWGSMSDATGRRPVFIGTFIVYLVANIALAESKNYGEL
MAFRALQAAGSAATISIGAGVIGDITNSEERGSLVGIFGGVRMLGQGIGPVFGGIFTQYLGYRSIFWFLTIAGGV
SLLSILVLLPETLRPIAGNGTVKLNGIHKPFIYTITGQTGVVEGAQPEAKKTKTSWKSVFAPLTFLVEKDVFITL
FFGSIVYTVWSMVTSSTTDLFSEVYGLSSLDIGLTFLGNGFGCMSGSYLVGYLMDYNHRLTEREYCEKHGYPAGT
RVNLKSHPDFPIEVARMRNTWWVIAIFIVTVALYGVSLRTHLAVPIILQYFIAFCSTGLFTINSALVIDLYPGAS
ASATAVNNLMRCLLGAGGVAIVQPILDALKPDYTFLLLAGITLVMTPLLYVEDRWGPGWRHARERRLKAKANGN

>A. niger_H915-1
MSSTTSSSRSDLEKVPVPQVTPRDSDSDKGSLSPEPSTLEAQSSEKPPHHIFTRSRKLQMVCIVSLAAIFSPLSS
NIYFPALDDVSKSLNISMSLATLTITVYMIVQGLAPSFWGSMSDATGRRPVFIGTFIVYLVANIALAESKNYGEL
MAFRALQAAGSAATISIGAGVIGDITNSEERGSLVGIFGGVRMLGQGIGPVFGGIFTQYLGYRSIFWFLTIAGGV
SLLSILVLLPETLRPIAGNGTVKLNGIHKPFIYTITGQTGVVEGAQPEAKKTKTSWKSVFAPLTFLVEKDVFITL
FFGSIVYTVWSMVTSSTTDLFSEVYGLSSLDIGLTFLGNGFGCMSGSYLVGYLMDYNHRLTEREYCEKHGYPAGT
RVNLKSHPDFPIEVARMRNTWWVIAIFIVTVALYGVSLRTHLAVPIILQYFIAFCSTGLFTINSALVIDLYPGAS
ASATAVNNLMRCLLGAGGVAIVQPILDALKPDYTFLLLAGITLVMTPLLYVEDRWGPGWRHARERRLKAKANGN

>A. oryzae
MATARSDTDKAPIVSFVEEDERQPSDSQLEDQVTQSKSHHIFSRRKKLQMVCIVSMAAIFSPLSSNIYFPALGEV
SRVSATKTKHSLCDLSRDVYMIVQGISPTFWGSISDATGRRPVFIGTFIVYMIANVALAVSTKYGELMAFRALQA
AGSAATISIGAGVIGDITTSAERGSLVGIFGGVRMLGQGIGPVMGGILTQYLGFRSIFWFLTICAAVSLLSILIF
LPETLRHIAGNGTVRLRGIHKPFLYVVIGQKGAITGADPGQKKPELTWRAILAPLTFLAEKDIFVTLLFGSIVYA

VWSMVTSSTTDLFQDVYHLTSLEVGLTFLGNGFGCISGSYLVGYLMDYNHKLTEREYCDKYGYPSGTRVNLKSHS
DFPIEVARMRHTWWIVGLFIVTTAVYGVSLRTHIAVPIILQYLIALCSTGIFTINSALVIDLYPGASASATAVNN
LIRCLVGAAGVAAMQPMLDVLTPDYVFLLLAGITLIMAPLLWMESRFGASWRHEREMRLKDKGCT

>A. terreus
MSSPDSTTHTPTTTPTVVTPDPPYSIFDKRQKWLIIIIVSTAATFSGFASNIYFPALPTIANDLNVSLELVNLTV
TSYLIFQGLAPSFWGPISDVKGRRTAYICTFIVFFCACIGLAETKNYATLIVVRCLQSAGSASTIAIGSGVIGDI
TTRADRGGYMGIFQAGLLVPVAVGPVIGGAIAGSLGWKAIFWFLAIYSGVFLCLLIAVLPETLRSIVGNGSRKSS
HPLRRYPLNLYQKSSKVPWQQGQDGQSTTAAKKIDLLGPLRMLLSNHAAPIIFFLAIYYAVWQMSITAMSSLFKT
RYGLSEIQIGLTFIANGVGSMVGTLVTGKILDADYRRVKTKYEASFDSEHGDAISQTAREENFPLESARLRLVPI
FSITQCLSIILFGWTIQYPNKVHIAVPIVSTFITGWTAVSTQSLIMTYLVDIFPDRSAAASASLNLARCLFAAGG
TSFIMPMINGVGVGVAFTICVVVQLVALIGPLVQWKFAARWRRKEREEAAQREEQKGEC

>A. tubingensis
MSSTTSSSRSDLEKVPVPQVTPRDNDSDKGSLSPEPSTLEAQSSEKPPHHIFTRSRKLQMVCIVSLAAIFSPLSS
NIYFPALDDVSKSLNISMSLATLTITVYMIVQGLAPSFWGSMSDATGRRPVFIGTFVVYLVANIALAESKNYGEL
MAFRALQAAGSAATISIGAGVIGDITNSEERGSLVGIFGGVRMLGQGIGPVFGGIFTQYLGYRSIFWFLTIAGGV
SLLSILVLLPETLRPIAGNGTVKLNGIHKPFIYSITGQKGVVEGAQPEAKKTQSSWKSVFAPLTFLVEKDVFITL
FFGSIVYTVWSMVTSSTTDLFSEVYGLSSLDIGLTFLGNGFGCMSGSYLVGYLMDYNHRLTEREYCEKHGYPAGT
RVNLKSHPDFPIEVARMRNTWWVIAIFIVTVALYGVSLRTHLAVPIILQYFIAFCSTGLFTINSALVIDLYPGAS
ASATAVNNLMRCLLGAGGVAVVQPILDALNPDYTFLLLAGITLVMTPLLYVEDRWGPGWRHARERRLKAKANGN

>A. versicolor
MAAKGPESTFEAVSSPAEAKPAPEIEPPYHILSKKQKWNLVIFVSLAGAFSPLSSNIYFPALDTISKDLGVSATL
TSLTITVYMIVQGIAPSLFGALSDSSGRRLTFTVSLTIYTAANLALAFTSNYAMLMVLRGVQAAGSAATISISAG
VIADIACPQERGGFMGTNAGIRMLGQGIGPVIGGLLNDAWGFRSIFWLLFVMSAIVLGALLVFLPETQRSRAGNG
SVPLSGFHKPLAYAFKPPMAWTQPSGTASPPKPHTPMNLKKAFSPLAYILEKDIATLLAWGAIAYTAWSMVTSST
TSMLLLGFPDLTQWQIGVCFLPNGVGCMAGSLSTGWLLDQGFRRAETNFKAKHGIAADEPIVPGAHGEEFSYIQA
RLRLMPLFSVVLVVSLALYGPSFEFNDARRYFAPNLAAPLVLQFLIAFTATSIFNINSTVLIDCFPDRPASATAL
NNLCRCLLGAAGVAAIEPLIGAVRAMRAFLIVTGIVVFFTPLIWVEWRFGEQWRKQRENRLAQAVA

>A. wentii
MDISKEKAPEATVTSLPGSRQTSPPESIKLEGQPEITPPPHHVFSRGQKLQMVYIVSLAAIFSPLSSNIYFPALG
DISKSLNTSMSLVTLTVTVYMIVQGLAPTFWGSMSDATGRRPVFIGTFAVYLIANIVLAVSTNYPELMVFRALQA
AGSAATISIGAGVIGDMTTSSERGSLVGIFGGVRMLGQGVGPVFGGILSEYLGFRSIFWFLTIAGAISLLSILVF
LPETLRPIAGNGSVPLTGFHKPFIYYIFPQKHAQEGATPGVEKTKVTWKAVFSPLTFLFEKDVFITLFFGSIVYT
FWSMVTSSTTSLFQEVYGLSTLQVGLTFLGNGFGCMSGSYSVGYLMDYNHRMTEREYCEKRGYPSGTRVNLKSHA
DFPIEVARMRNTWWVIALFVITTALYGVSLRTHLAVPIILQYFIAFCATGIFTINSAFVIDLYPGASASATAVNN
LIRCLIGAAGVAVVQPIIDALTAEYTFIMLAGITVGMTPLLWIESKYGPGWRHAREERLARK

>A. fischeri
MSGGVREIQERVAPSSSSNDSENVIQYHVEKPAEVSSGSDPSALEAQKATTLQADGETDGEAHSAFSKNEKRFIV
FMISMASFFSPLSAQIYFPVMPTLVKNYHLTTALINLTITTYLILQGLAPSFIGTFADSGGRRPAYILAFTVYLA
ANIGLALQNSYVALMVLRCIQSAGSSGTIAFGYGVVADIATAAERGSFIGPVSAGVMVAPALGPVIGGILAKFLG
WRSVFWFLVIISGSYLVVFMITMPETNRNIVGNGSVPPDQWWRMSVIQYLAARRRLQKMTAEERLAHQEQRANLN
QAREHQKLSFPNPLKAFAILLEKDAFIVVSYVGLAMLANTALLTSTPNLFGKLYGFNDLQIGLCYIPLGVGSCIA
AILNGKLLDFNYRRIAHKIGMPVDRKKGDDLRGFPIEKARLQAFFPLMALGVAAFIPYGWVLQQGAPLAAPLVLQ
FIIGFSFIASLNTLNTLMVDLFPDRASTASAASNLVRCWLGAVGAAVIDHMLSAMGWGWCFVFWGLLMLVGLGLL
WVEYHRGMKWRLARLTKLDQKRVEKETQKAASENKEAHGDENEANSDT

>A. niger_ATCC64974_SBP43542
MVSVKSSEKQCDDPDPKAERVPSDTEPPQEDPNSTPGETEVYSTFSRWTKALIVLMASVASIFSSLSANIYLPAL
DTIAEQLHVSDTLINLTLSTYMIFQGLAPTIFGGLSDSAGRRPAYILCFITYIAANIGLALQRNYAALLILRCLQ
SAGGSSTIALANAIVADVVTTAERGRYIGYVSAGFILGPSMGPIIGGLLTQFLGWPFIFWLLTILAGAFFIVLLF
FLPETARKVVGNGSQYPPIWNLSGLQLCSRRQRADGYSSVETHIRFPNPLKALTIIFKKEVFLILLGNGVAIAGY
FAVTGTITSQFSRIYGFNEVQIGLCFIPLGAGNLIAAYTQGIITDWNYRRHAHSQGIDISSKHRPDLDKFAIERP
RTEVVLPQAIGEALSMIAYGWVLHYETNLAGPIILLLLIGYTSAAVMNTLSALMVDIYPQSPAMATALMNLTRCW
LGAGAAAAILPMIDAIGNGWAFTVIGFMCFLCAPAMVVVWIWGYDWRRQRHCREETSLGNHISN

>A. niger_ATCC64974_SPB46513
MPSEDSATPVQSNTSTGVATPEPPYSIFDKRQKWLIIIIVSTAATFSGFASNIYFPALPTIANDLNVSIELLNLT
VTSYLIFQGLAPSLWGPVSDVKGRRTAYMCTFVVFLGACIGLAESKNYATLIILRCLQSTGSASTIAIGSGVISD
ITTRAERGGYMGIFQAGLLVPVAVGPVIGGGIAGSLGWKAIFWFLTIYSGAFLCFLIVFLPETLRSIVGNGSLKS
SNPLVNFPLELYQKTTKVEWEQVPDGQSQRAAKKKIDVLGPLRILISNHAAPIIIFLAVYYAVWQMSITAMSSLF
KSRYGLSEIQIGLTFIANGVGSMIGTLVTGKILDADYRRVKSRYEASLDAEQGEETTAATREENFPLEKARLRLV
PIFSILQCLSIILFGWTINYARKVHIAVPIVSTFITGWTAVSTQSLIMTYLVDIFPDRSAAASASLNLARCLFAA
GGTSFIMPLINSVGVGVAFTICVAVQLLALVGPLIQWRFAAGWRRKERERDAQRADVKN
