# Supplementary material for: Global Transcriptional Response of Aspergillus niger to Blocked Active Citrate Export through Deletion of the Exporter Gene
Source: J Fungi (Basel). 2021 May 23;7(6):409. doi: 10.3390/jof7060409 (PMC8224569; doi:10.3390/jof7060409)
Supplement: Supplementary file 1 [file jof-07-00409-s001.zip › Supplementary Table S1_Primers.pdf]

Global transcriptional response of *Aspergillus niger* to blocked active citrate export through deletion of the exporter gene

Thanaporn Laothanachareon[1,2,a,*], Lyon Bruinsma[1], Bart Nijsse[1], Tom Schonewille[1], Maria Suarez Diez[1], Juan Antonio Tamayo-Ramos[3], Vitor AP Martins dos Santos[1,4,*#], Peter J. Schaap[1#]

[1] Laboratory of Systems and Synthetic Biology, Wageningen University & Research, Wageningen, The Netherlands

[2] Enzyme Technology Laboratory, Biorefinery and Bioproduct Research Group, National Center for Genetic Engineering and Biotechnology, 113 Thailand Science Park, Khlong Luang, Pathumthani 12120, Thailand

[3] International Research Center in Critical Raw Materials-ICCRAM, University of Burgos, Burgos, Spain.

[4]LifeGlimmer GmbH, Berlin, Germany

# Joint senior authors

*** Corresponding author:**

Thanaporn Laothanachareon

Mailing address: Enzyme Technology Laboratory, Biorefinery and Bioproduct Research Group, National Center for Genetic Engineering and Biotechnology, 113 Thailand Science Park, Khlong Luang, Pathumthani 12120, Thailand

Email: thanaporn.lao@biotec.or.th (TL)

Vitor AP Martins dos Santos

Mailing address: Laboratory of Systems and Synthetic Biology, Wageningen University & Research, Wageningen, The Netherlands

Email: vitor.martinsdossantos@wur.nl (VAPMS)

[a]Current address: Enzyme Technology Laboratory, Biorefinery and Bioproduct Technology Research Group, National Center for Genetic Engineering and Biotechnology, 113 Thailand Science Park, Khlong Luang, Patumthani 12120, Thailand

Supplementary Table S1 | Primers used in this study

Primer name	Sequence 5' - 3'
citT_1FW	ATGTCTTCAACCACGTCTTCATC
citT_1770RV	CTAGTTGCCGTTGGCTTTGG
citT_KO_5Flank_FW_p1	TGAGCAGTTCGCCAATCAGG
citT_KO_5Flank_RV_p2	CAATTCCAGCAGCGGCTTGATTGTGGATATGACTCGGG
citT_KO_3Flank_FW_p3	ACACGGCACAATTATCCATCGGGAGAGAAAGGACTTGAAAAAAAAAGG
citT_KO_3Flank_RV_p4	AGCTTCTTACCCATCTCAGG
citT_KO_FW_p11	TATGGGCCTGATGTAGATCC
citT_KO_RV_p12	TCAGATGGAAGCAGACACGTCG
AOpyrG12FW_p5	AAGCCGCTGCTGGAATTG
AOpyrG13FW_p6	CGATGGATAATTGTGCCGTGT
AOpyrG14FW_p7	ATTGACCTACAGCGCACGC
AOpyrG15RV_p8	CCGGTAGCCAAAGATCCCTT
AOpyrG_KO _RV_p13	GGCATTTGTCGTGCAGTTTG
AOpyrG_KO _FW_p14	AATTCCACGGCCAGCATTAA
