# Supplementary material for: Global Transcriptional Response of Aspergillus niger to Blocked Active Citrate Export through Deletion of the Exporter Gene
Source: J Fungi (Basel). 2021 May 23;7(6):409. doi: 10.3390/jof7060409 (PMC8224569; doi:10.3390/jof7060409)
Supplement: Supplementary file 1 [file jof-07-00409-s001.zip › Supplementary Table S2_CDW.pdf]

Global transcriptional response of *Aspergillus niger* to blocked active citrate export through deletion of the exporter gene

Thanaporn Laothanachareon[1,2,a,*], Lyon Bruinsma[1], Bart Nijsse[1], Tom Schonewille[1], Maria Suarez Diez[1], Juan Antonio Tamayo-Ramos[3], Vitor AP Martins dos Santos[1,4,*#], Peter J. Schaap[1#]

[1] Laboratory of Systems and Synthetic Biology, Wageningen University & Research, Wageningen, The Netherlands

[2] Enzyme Technology Laboratory, Biorefinery and Bioproduct Research Group, National Center for Genetic Engineering and Biotechnology, 113 Thailand Science Park, Khlong Luang, Pathumthani 12120, Thailand

[3] International Research Center in Critical Raw Materials-ICCRAM, University of Burgos, Burgos, Spain.

[4]LifeGlimmer GmbH, Berlin, Germany

# Joint senior authors

*** Corresponding author:**

Thanaporn Laothanachareon

Mailing address: Enzyme Technology Laboratory, Biorefinery and Bioproduct Research Group, National Center for Genetic Engineering and Biotechnology, 113 Thailand Science Park, Khlong Luang, Pathumthani 12120, Thailand

Email: thanaporn.lao@biotec.or.th (TL)

Vitor AP Martins dos Santos

Mailing address: Laboratory of Systems and Synthetic Biology, Wageningen University & Research, Wageningen, The Netherlands

Email: vitor.martinsdossantos@wur.nl (VAPMS)

[a]Current address: Enzyme Technology Laboratory, Biorefinery and Bioproduct Technology Research Group, National Center for Genetic Engineering and Biotechnology, 113 Thailand Science Park, Khlong Luang, Patumthani 12120, Thailand

Supplementary Table S2 | Cell dry weight of *A. niger* strain N402 and *ΔcitT* grown on various carbon sources by fermentation

A. Cell dry weight of *A. niger* strain N402 and *ΔcitT* grown on glucose medium by fermentation

Strain	T_0			Average
	1	2	3	
N402	0.9301	0.8618	0.9318	0.9080 ± 0.0400
ΔcitT1.1	0.9507	0.9509	0.9530	0.9520 ± 0.0010
**Strain**	T_{216}			**Average**
	**1**	**2**	**3**	
N402	14.7673	15.9784	16.3066	15.6840 ± 0.8110
ΔcitT1.1	15.0927	16.0831	14.8003	15.3250 ± 0.6720

B. Cell dry weight of *A. niger* strain N402 and *ΔcitT* grown in citrate medium by fermentation

Strain	T_0			Average
	1	2	3	
N402	1.0881	1.0732	1.0807	1.0807 ± 0.0075
ΔcitT1.1	1.0610	0.9848	0.9905	1.0121 ± 0.0424
**Strain**	T_{72}			**Average**
	**1**	**2**	**3**	
N402	1.4450	1.4080	1.3145	1.3892 ± 0.0673
ΔcitT1.1	1.7934	1.3243	1.3562	1.4913 ± 0.2621
